# Contrast Agents Based on Human Serum Albumin and Nitroxides for ^1^H-MRI and Overhauser-Enhanced MRI

**DOI:** 10.3390/ijms25074041

**Published:** 2024-04-05

**Authors:** Dmitry Mitin, Friedemann Bullinger, Sergey Dobrynin, Jörn Engelmann, Klaus Scheffler, Mikhail Kolokolov, Olesya Krumkacheva, Kai Buckenmaier, Igor Kirilyuk, Alexey Chubarov

**Affiliations:** 1 Institute of Chemical Biology and Fundamental Medicine SB RAS, 630090 Novosibirsk, Russia; d.mitin@g.nsu.ru; 2 High-Field Magnetic Resonance Center, Max Planck Institute for Biological Cybernetics, 72076 Tuebingen, Germany; friedemann.bullinger@tuebingen.mpg.de (F.B.); joern.engelmann@tuebingen.mpg.de (J.E.); klaus.scheffler@tuebingen.mpg.de (K.S.); kai.buckenmaier@tuebingen.mpg.de (K.B.); 3 N.N. Vorozhtsov Institute of Organic Chemistry SB RAS, 630090 Novosibirsk, Russia; s.a.dobrynin@gmail.com; 4 Department of Biomedical Magnetic Resonance, Eberhard-Karls University, 72076 Tuebingen, Germany; 5 International Tomography Center SB RAS, 630090 Novosibirsk, Russia; m.kolokolov@tomo.nsc.ru (M.K.); olesya@tomo.nsc.ru (O.K.)

**Keywords:** nitroxides, spin probes, organic radical contrast agents, human serum albumin, magnetic resonance imaging, Overhauser dynamic nuclear polarization (ODNP) enhanced magnetic resonance imaging (OMRI)

## Abstract

In cancer diagnostics, magnetic resonance imaging (MRI) uses contrast agents to enhance the distinction between the target tissue and background. Several promising approaches have been developed to increase MRI sensitivity, one of which is Overhauser dynamic nuclear polarization (ODNP)-enhanced MRI (OMRI). In this study, a macromolecular construct based on human serum albumin and nitroxyl radicals (HSA-NIT) was developed using a new synthesis method that significantly increased the modification to 21 nitroxide residues per protein. This was confirmed by electron paramagnetic resonance (EPR) spectroscopy and matrix-assisted laser desorption/ionization time-of-flight (MALDI ToF) mass spectrometry. Gel electrophoresis and circular dichroism showed no significant changes in the structure of HSA-NITs, and no oligomers were formed during modification. The cytotoxicity of HSA-NITs was comparable to that of native albumin. HSA-NITs were evaluated as potential “metal-free” organic radical relaxation-based contrast agents for ^1^H-MRI and as hyperpolarizing contrast agents for OMRI. Relaxivities (longitudinal and transversal relaxation rates *r*_1_ and *r*_2_) for HSA-NITs were measured at different magnetic field strengths (1.88, 3, 7, and 14 T). Phantoms were used to demonstrate the potential use of HSA-NIT as a *T*_1_- and *T*_2_-weighted relaxation-based contrast agent at 3 T and 14 T. The efficacy of ^1^H Overhauser dynamic nuclear polarization (ODNP) in liquids at an ultralow magnetic field (ULF, *B*_0_ = 92 ± 0.8 μT) was investigated for HSA-NIT conjugates. The HSA-NITs themselves did not show ODNP enhancement; however, under the proteolysis conditions simulating cancer tissue, HSA-NIT conjugates were cleaved into lower-molecular-weight (MW) protein fragments that activate ODNP capabilities, resulting in a maximum achievable enhancement |*E*_max_| of 40–50 and a radiofrequency power required to achieve half of *E*_max_, *P*_1/2_, of 21–27 W. The HSA-NIT with a higher degree of modification released increased the number of spin probes upon biodegradation, which significantly enhanced the Overhauser effect. Thus, HSA-NITs may represent a new class of MRI relaxation-based contrast agents as well as novel cleavable conjugates for use as hyperpolarizing contrast agents (HCAs) in OMRI.

## 1. Introduction

Magnetic resonance imaging (MRI) is one of the most common medical techniques for obtaining high-quality three-dimensional soft tissue images [1,2,3,4,5,6]. ^1^H-MRI provides information about various biological processes, the diagnosis of diseases, and monitoring therapy efficiency [2,3,6]. MRI is typically performed for water protons. The application of external magnetic field leads to the relatively low thermal equilibrium spin polarization of protons (approximately 0.0003% per Tesla, at body temperature) [7]. Due to the low polarization and relatively slow relaxation of nuclei, magnetic resonance signals are weak, and MRI usually requires strong magnetic fields and lengthy acquisition times.

The most common way to increase MRI sensitivity is an enhancement of the spin relaxation of protons by the addition of a contrast agent (CA). Paramagnetic Gd^3+^ and Mn^2+^ complexes, and magnetite nanocomposites, are among the most widely used CA for ^1^H-MRI [2,5,8,9,10,11,12,13,14]. However, in recent decades, the European Medicines Agency and U.S. Food and Drug Administration (FDA) have prohibited the use of many Gd^3+^ complexes and magnetite nanoparticles for MRI in clinics [15] due to their side effects and toxicity, including reactive oxygen species generation, neurotoxicity, mitochondrial dysfunction, nucleic acid damage, nephrogenic systemic fibrosis (NSF), etc. [8,9,16,17,18,19,20]. Therefore, there is an extensive interest in the development of “metal-free” CAs.

Stable organic radicals are considered an alternative to paramagnetic metal complexes such as CA for MRI [21]. Organic radical CAs (ORCAs) are compatible with standard imaging protocols, biocompatible, and biodegradable [21,22,23,24,25,26,27]. Although low-molecular radicals demonstrate low relaxivity, fast decay in vivo and are rapidly eliminated [22,28,29,30,31,32,33], the incorporation of multiple radicals into macromolecular or supramolecular structures afforded ORCA, capable of competing with Gd complexes [23,24,25,26,28,33,34,35]. Various nitroxide-based systems, such as encapsulated micelles [36], functionalized polymers [26,27,37], dendrimers [24,25,26,38,39,40,41], and proteins [23], have been developed for ^1^H-MRI.

Numerous methods are currently being developed to increase the sensitivity of MRI. One effective approach is the use of the dynamic nuclear polarization (DNP) effect at high magnetic fields (>1 T) and temperatures below 5 K [42]. This commercially available technique (dissolution DNP) allows the hyperpolarization of substances such as pyruvate in the form of glassy samples in the solid state, achieving spin polarization levels of up to 80%. After the rapid heating and dissolution of the sample to the liquid state, the resulting hyperpolarized substrate can be introduced into the sample as a biocompatible bolus. One alternative promising avenue is Overhauser dynamic nuclear polarization (ODNP)-enhanced MRI (OMRI). Unlike other dynamic nuclear polarization (DNP) methods that rely on the cross or solid effect, or thermal mixing, OMRI exploits the Overhauser effect [43,44,45].

The Overhauser effect relies on incoherent electron–nuclear cross-relaxation mediated by dipole, scalar, or isotropic hyperfine interactions [14]. Unlike thermal mixing or the solid or cross effect, the Overhauser effect allows the hyperpolarization of liquids at room temperature [46]. This unique advantage allows for continuous in situ, ex vivo, or even in vivo hyperpolarization of the sample itself, eliminating the need for a hyperpolarized bolus. However, ODNP typically results in smaller enhancement factors, of less than 100 for ^1^H in vivo OMRI applications. Biomedical applications of OMRI include the measurement of tissue oxygen partial pressure, pH, redox status, enzymatic activity, etc. [47,48,49,50,51].

All DNP methods share the common requirement to transfer spin order from free electrons to nearby protons, such as those in water molecules, which requires an RF field with a frequency in the range of the electron Larmor frequency. The latter exceeds the GHz range at the magnetic field of approximately 36 mT (penetration depth around 1 GHz is only a few centimeters, above 10 GHz the penetration depth is less than 1 mm). RF of this frequency is efficiently absorbed by biological tissues, therefore penetration deep into larger samples becomes challenging. Exposure to high-power RF may lead to the overheating of biological tissue. For this reason, in vivo OMRI is generally performed in the low field range, with the optimal field strength depending on the sample geometry and the specific absorption rate (SAR), which must be considered to prevent sample heating [44,52,53]. Nevertheless, in vivo experiments on small animals have been performed using OMRI [46,48]. The success of OMRI depends on the presence of special hyperpolarizing contrast agents (HCAs) that facilitate the transfer of polarization from free electrons, such as free radicals, to nuclear spins, such as water protons, thereby enhancing the MRI signal [14,46]. It is noteworthy that this method is not limited to protons, as the hyperpolarization of other MR-active nuclei, such as ^13^C, is also possible [54]. Several research groups have already performed OMRI experiments in liquids or small animals using trityl [55] or nitroxyl radicals [46,53,56,57]. Furthermore, radical molecules can be attached to various transport systems for targeted delivery, thereby increasing their half-life in the body.

Here we develop a class of macromolecular CA based on nitroxides (NIT) and human serum albumin (HSA) for ^1^H-MRI, which also can be used as an HCA for OMRI. Albumin is a major plasma transport protein, which allows accumulation in tumors via passive transport (enhanced permeability and retention effect) or via albumin-binding proteins and receptors [58], making it useful as a delivery system for therapy and diagnostics [8,23,59,60,61,62,63]. HSA as a probe carrier has high biocompatibility and retention time in the body, low immunogenicity, and structural stability toward chemical modification [8,64,65,66,67,68,69,70,71,72,73,74].

These new constructs (HSA-NIT) retain the essential properties of nitroxides, such as paramagnetism, which was demonstrated by room-temperature continuous-wave electronic magnetic resonance (CW EPR) and relaxivities r_1_ and r_2_ under various magnetic field studies. To confirm the possibility of MRI signal registration, phantom *T*_1_- and *T*_2_-relaxation-weighted images were obtained using 3 T and 14 T MRI scanners. HSA-NIT retains the properties of albumin such as low cytotoxicity on breast cancer MCF-7 and human glioblastoma T98G cell cultures using an MTT assay [75,76]. The comprehensive characterization of HSA-NIT conjugates was facilitated by MALDI ToF mass spectrometry, polyacrylamide gel electrophoresis in native (PAGE) and denaturation condition (SDS-PAGE), and circular dichroism (CD) spectroscopy. To confirm the possibility of OMRI signal registration, we digested HSA-NIT using trypsin and studied the efficacy in ^1^H Overhauser DNP (ODNP) in aqueous solution in an ultralow magnetic field (ULF, *B*_0_ = 92 ± 0.8 μT) and polarization field (*B_p_* = 1–10 mT) per time. Under protein proteolysis, which can occur in cells and cancer tissues, EPR spectra contain narrow spectral lines, and highly efficient ODNP enhancement can occur. The results from HSA-NIT studies have shown great promise for dual-modal MRI and enzyme-activated OMRI probe design.

## 2. Results and Discussion

### 2.1. Design and Synthesis of HSA-NIT Conjugates

**Figure 1:**
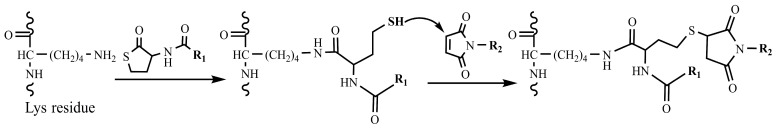
Protein acylation by HTL with subsequent reaction with a maleimide derivative.

Site-specific labeling is most commonly performed on cysteine [23,77,78], lysine [23,79], and tyrosine [77,80] in proteins. However, for MRI applications, many nitroxide residues should be attached to HSA. In this way, the modification of amino groups of lysine seems to be the optimal choice due to the presence of 59 lysine amino acid residues in albumin molecule. Previously, we synthesized albumin conjugates with three to four nitroxides (HSA-NIT) via a site-specific reaction with *N*-spin-labeled homocysteine thiolactone (HTL) [23]. HTL has a strained five-membered ring, which can react with various nucleophiles (e.g., amino group of lysine residues) [81], leading to ring opening and stable amide bond formation (Figure 1). *N*-Homocysteinylation is a site-specific reaction that proceeds on seven lysine residues (Lys-525, 212, 205, 159, 137, 12, and 4) [23,63,82,83,84], and it is possible to vary the extent of modification by changing the reaction conditions and obtain new conjugates with specified properties. HSA-NIT conjugates provided good stability, relative to gadolinium chelates relaxivity, and there was no cytotoxicity [23]. However, the homocysteinylation reaction leads to the release of thiol groups in the side chain, which may initiate structural changes in the protein and oligomerization via thiol exchange and oxidation to disulfides.

**Figure 2:**
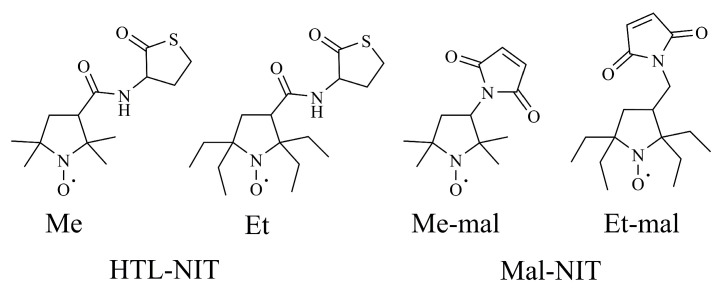
Homocysteine thiolactone and maleimide nitroxide derivatives.

Herein, we used both thiolactone (HTL-NIT) and maleimide (Mal-NIT) nitroxide derivatives for HSA modification (Figure 2). Maleimides are well-known reagents that rapidly bind SH-groups via the Michael addition reaction [82]. In this alternative method, the SH-groups released in the reaction of lysine residues and HTL-NIT can react with the Mal-NIT, providing the double spin labeling of a single lysine residue in a protein (Figure 1) [85]. This double modification allows the prevention of secondary reactions of SH-groups, which can damage the protein structure.

### 2.2. HSA-NIT Synthesis Method with Sequential and Simultaneous Addition of NIT Derivatives

**Figure 3:**
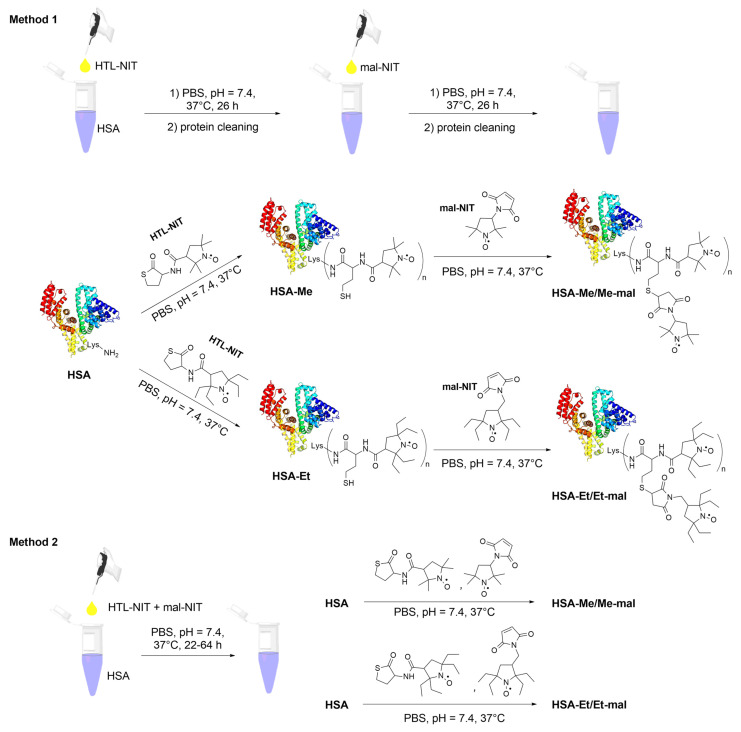
HSA-NIT conjugate synthesis schemes. HSA is shown schematically as a helical ribbon structure.

Two different sets of tetramethyl- (Me) or tetraethyl (Et)-substituted nitroxides were attached to HTL or Mal (Figure 2) and different procedures for the preparation of spin-labeled HSA were compared. The first method we used for conjugate synthesis was albumin acylation by a 30-fold excess of HTL-NIT, with subsequent protein cleaning using Centricon concentrators and PBS as eluent (Figure 3, Method 1).

In the second stage, a 15-fold excess of Mal-NIT compounds was added to HSA-Me and HSA-Et solutions. At each stage, the synthesis was analyzed via gel electrophoresis (SDS-PAGE) and CW EPR. To the HSA-Me and HSA-Et, on average, 4.8 and 5.2 nitroxide residues were attached, respectively, which was calculated by CW EPR. For HSA-Me/Me-mal and HSA-Et/Et-mal conjugates, the average modification degrees are equal to 7.0 and 10.2, which are double those obtained in the HSA-NIT in our group [23].

**Figure 4:**
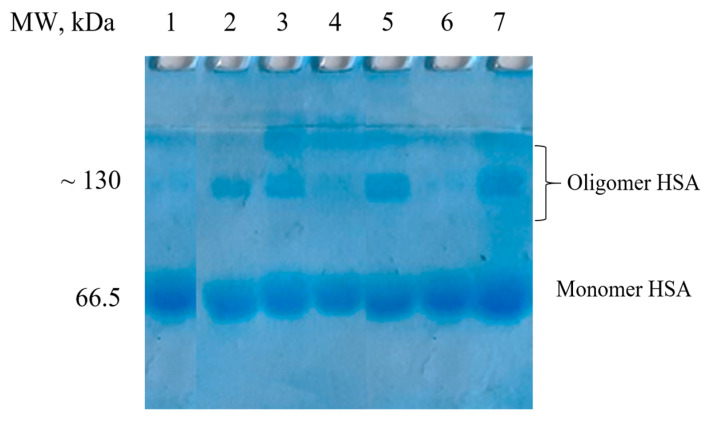
SDS-PAGE of HSA-NIT under Laemmli conditions with subsequent Coomassie blue staining. Control monomer fraction of HSA (lane 1). Method 1 synthesis: HSA-Et (lane 2), HSA-Me (lane 3), HSA-Me/Me-mal (lane 5), HSA-Et/Et-mal (lane 7). Method 2: HSA-Me/Me-mal (lane 4), HSA-Et/Et-mal (lane 6).

The bulk structure of HSA can change during modification, which can lead to conformational changes, aggregation, and the formation of potentially toxic oligomers [86,87]. According to SDS-PAGE data (Figure 4), the protein oligomer content in HSA-Me and HSA-Et was 11–12%. New SH-groups introduced in HSA by the reaction with HTL-NIT were partly oxidized, resulting in HSA-S-S-HSA oligomers, as was shown by SDS-PAGE in the presence of the reduction agent dithiothreitol (DTT). Such oligomer formation may be an artifact of the use of DMSO, which is known as a mild oxidizing agent for SH groups [88,89]. Nevertheless, DMSO is a widely used biocompatible solvent; it can cause protein three-dimensional structure disruption, α-helical and β-sheet content changes, oligomerization, and high excess precipitation [23,90]. In this way, we have used as low a dose of DMSO as possible (5%) in the solution for nitroxides poorly soluble in water, which, however, did not stop albumin oligomerization (Figure 4). For HSA-Me/Me-mal and HSA-Et/Et-mal, the oligomers’ contents doubled with 17–20%.

To reduce the amount of oligomers, present in the synthesis of HSA-NIT, it was proposed to carry out the reactions via the simultaneous addition of HTL and maleimide NIT derivatives (Figure 3, Method 2). To compare Method 1 and 2, we used the same reaction conditions, such as 30- and 15-fold excesses of HTL-NIT and Mal-NIT, respectively. Under these conditions, no oligomers were found for HSA-NIT conjugates in Method 2 (Figure 4, lanes 4 and 6). However, fewer nitroxides were attached to albumin (Table S1, e.g., for HSA-Et/Et-mal, 10 and 8 for Methods 1 and 2, respectively). Further analysis shows that the simultaneous presence of two nitroxide derivatives in the tube leads to the precipitation of some compounds due to their low solubility in aqueous solution [91]. We tried to change protein concentration, reagent excess, and reaction time, which allowed us to increase the degree of modification to ~14 nitroxides (Table S1). We are faced with the insurmountable problem of the low solubility of nitroxides in water and the inability to increase the amount of DMSO due to the influence of a high DMSO concentration on the protein structure. An amount of DMSO of more than 15% leads to a decrease in α-helical content with full precipitation of the protein in 40% DMSO solution.

**Figure 5:**
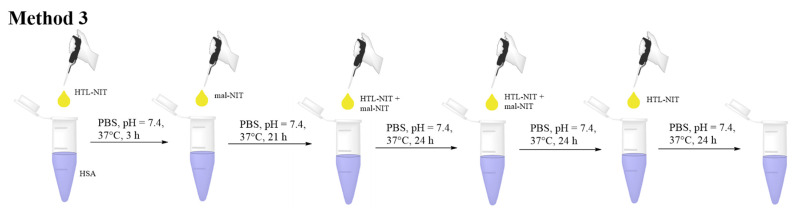
Method 3 of HSA-Me/Me-mal and HSA-Et/Et-mal synthesis.

To solve the problem, we adapted Method 2 by adding NIT derivatives in small portions at equal time intervals (Method 3, Figure 5, Table S2). First of all, we mixed the aqueous solution of albumin with a small amount of DMSO, leading to a 7% DMSO solution. Then, the portion of HTL-NIT derivative was added to synthesize the initial amount of HSA-Me and HSA-Et with a free SH-group for further reaction with Mal-NIT. Although maleimides have high thiol specificity under high excess and/or basic pH, they can react with amino acid residues such as lysine, imidazole, and histidine [85,92,93]. To avoid a non-specific reaction, maleimide was added with a 3 h time delay (Figure 5). In the next stages, we added maleimide and HTL derivatives together. This partial, sparing addition of soluble NIT maintains a consistently high concentration in solution in relatively low excess. In such conditions, the first portions of nitroxide derivatives are not precipitated at all, which allows for significant savings in reagents. The yields of HSA-Me/Me-mal and HSA-Et/Et-mal were approximately 85% and 84%, respectively. The HSA-NIT modifications were proven by CW EPR (Figure S1) and MALDI-ToF MS (Figure S2), which showed 17 and 21 nitroxide residues for HSA-Me/Me-mal and HSA-Et/Et-mal, respectively. For comparison, we have synthesized HSA-Me and HSA-Et samples by protein acylation using HTL-NIT derivatives (Table S2). As the modification of HSA-NIT increased (e.g., cf. HSA-Me and HSA-Me/Me-mal), there was a change in the UV spectrum, leading to the disappearance of the distinct absorption maximum of HSA at 278 nm and the minimum at ~253 nm (Figure S3). This effect is caused by the presence of NIT absorption at 240–270 nm. For the comparison and investigation of properties, we have synthesized HSA-Me-mal and HSA-Et-mal samples (Table S2). HSA-Me-mal and HSA-Et-mal conjugates were synthesized by an albumin reaction with pure maleimide derivatives. Interestingly, the high modification degree of 16 and 22 nitroxides was observed for HSA-Me-mal and HSA-Et-mal, respectively, which affirmed the possibility of a non-specific reaction with the protein’s amino acids.

### 2.3. Characterization of HSA-NIT Conjugates Synthesized by Method 3 Using SDS-PAGE, MALDI-ToF, Circular Dichroism, and EPR

For the synthesis of conjugates by Method 3, we used not a monomer, but a full HSA fraction, with 4% of oligomers (see SDS-PAGE, Figure S4). For HSA-Me and HSA-Et samples, we observed a slight increase in oligomers from 4% to 9% (Figure S4). Conjugates HSA-Me/Me-mal and HSA-Et/Et-mal showed no significant changes from the starting HSA. For extra HSA-NIT characterization, native PAGE with SDS was performed (Figure S5). Native gel electrophoresis can easily provide information about changes in conformation and charge. HSA-NIT modification leads to bands broadening and increasing their electrophoretic mobility compared to native HSA. Moreover, the mobility of HSA-Me/Me-mal and HSA-Et/Et-mal bands is much higher than for HSA-Me and HSA-Et, which can be explained by a lower negative charge of the latter ones. Albumin modification by HTL-NIT enables the disappearance of the positive charge of the ɛ-amino group of lysine to a neutral charge, which leads to an increase in protein negative charge, leading to higher gel electrophoretic mobility.

All samples were analyzed for protein stability by SDS-PAGE for one year. We provide SDS-PAGE after the synthesis (Figure S4), after 3 months, and after 1 year of sample storage in PBS solution at 4 °C (Figure S6). HSA-NIT conjugates were stable for 3 months without any changes in band quality. After one year, some protein fragmentation was observed with ~55 and ~45 kDa bands formation (Figure S6b). The amount of protein fragments was ~5% for HSA-Me and -Et and 15–20% for other conjugates with high nitroxide modification (HSA-Me/Me-mal, HSA-Et/Et-mal, HSA-Me-mal, and HSA-Et-mal) (Figure S6b).

Changes in the three-dimensional structure of HSA-NIT were analyzed by circular dichroism (CD). From CD spectra, α-helices and β-sheets were calculated (Table 1) by the deconvolution of CD spectra using theoretical curves [94,95]. The α-helices decreased from 55% to 48–49% for slightly modified HSA-Me and -Et conjugates. For HSA-Et-mal and HSA-Et/Et-mal, a significant reduction in α-helices to ~43–44% with a slight increase in β-sheets by 3–4% was observed. Protein over-labeling usually leads to significant changes in conformation. In this way, the coupling of up to ~20 molecules without oligomer formation and only moderate secondary structure changes are good results of protein modification.

HSA-NIT conjugates were analyzed by CW EPR. The attachment of a nitroxide to albumin significantly increases its rotation correlation time, leading to line broadening (Figure S1) [23,59]. Using EasySpin software 5.2.35, experimental EPR spectra were separated into three components with different rotational correlation times (τ_c_)—“slow”, “fast” and “free” (Table S3). For example, in the EPR spectrum of HSA-Me, broad (~71%) and narrow (~29%) components, with τ_c_ of 8.6 ns (“slow”) and 1.1 ns (“fast”), respectively, can be easily seen. A similar behavior was obtained for HSA-Me/Me-mal, HSA-Et, and HSA-Et/Et-mal, with τ_c_ of 7.3–8.6 ns and 0.5–0.9 ns. Most nitroxides have long τ_c_ and therefore “slow” behavior, which indicates localization in a non-polar environment inside pockets of the protein [95].

### 2.4. Reduction Constant Calculation of HSA-NIT

The bioreduction of nitroxides to diamagnetic compounds is a well-known problem encountered in radical-based MRI contrast agent design [23,26,38,96,97]. We evaluated the reduction constants of HSA-NIT conjugates using biogenic reducing agents, such as ascorbic acid and glutathione [23,98,99]. Reduction rates were measured under pseudo-first-order conditions using a high excess of reducing agents in PBS (pH 7.4). We obtained an effective constant, which was recalculated into second-order rate constants (Table 2). Two methods were used for experimental decay analysis, such as CW EPR and T_1_ relaxation time measurement [23]. We found that attachment to the albumin significantly decreases the reduction constant (cf. free radical and attached, Table 2), which is especially pronounced for Me radicals.

The protein environment alters the reduction constant for “slow” radicals, resulting in their protection from reducing agents. The reduction resistance of nitroxides in HSA-NIT is an essential feature for further MRI applications. The decrease in the reduction constant occurs due to an increase in the proportion of “slow” NIT with the greater modification of HSA-NIT. The lowest reduction rate constant, k = 0.0007 M^−1^s^−1^, was obtained by measuring T_1_ for HSA-Et/Et-mal, which is most deeply modified with nitroxides (~21 nitroxides per molecule). This value is 2.5 times lower than for a similar conjugate obtained previously (k = 0.0018 [23]) with only four nitroxides and an order of magnitude lower than dendrimers-based ORCAs (k = 0.0376 [26]). Thus, albumin-based conjugates with sterically hindered tetraethyl-substituted nitroxide derivatives can maintain the paramagnetic properties of NIT in vivo for a long time.

### 2.5. Cytotoxicity Assay

**Figure 6:**
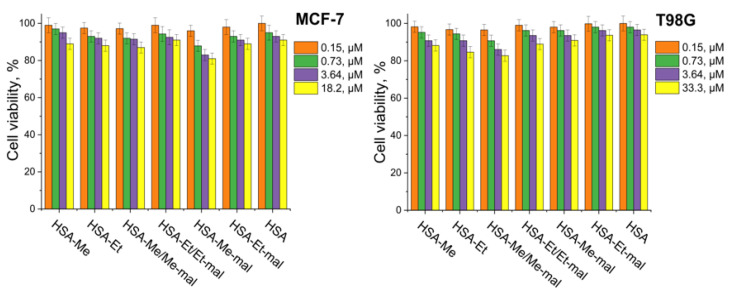
Cytotoxicity assay on MCF-7 and T98G cell cultures for HSA-NIT conjugates. Cell viability was normalized using cells treated with PBS buffer as a 100% viability control.

The colorimetric method using the 3-(4,5-dimethylthiazol-2-yl)-2,5-diphenyltetrazolium bromide (MTT) compound was provided to characterize cellular viability in the presence of HSA-NIT [75,102]. The MTT test was performed on breast cancer MCF-7 and human glioblastoma T98G cell cultures using the standard procedure. Increasing the concentration of conjugates in T98G and MCF-7 cell culture medium from 0.15 μM to 18.20 μM decreased viability for native HSA and HSA-NIT conjugates by similar values (Figure 6), which indicates their low cytotoxicity.

### 2.6. Relaxaxivities r_1_ and r_2_ of HSA-NIT

**Figure 7:**
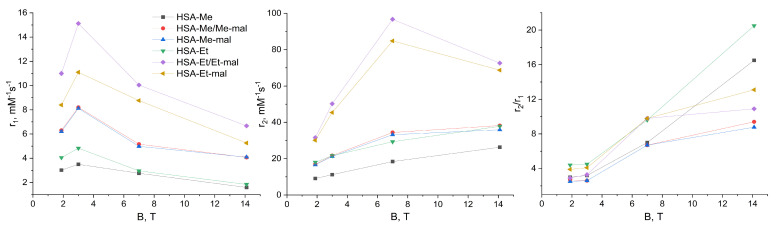
Relaxivities *r*_1_ and *r*_2_ (per protein molecule concentration, mM^−1^s^−1^), and *r*_2_/*r*_1_ ratio for HSA-NIT conjugates per magnetic field. The legend for three pictures is presented on the left.

Relaxivities *r*_1_ and *r*_2_ highly influence the level of sensitivity in the registration of *T*_1_- and *T*_2_-weighted MRI. The higher relaxivity indicates a higher contrast of the image. One nitroxide has a low relaxivity due to the presence of only one unpaired electron. However, HSA-NIT has multiple nitroxides per albumin molecule (Table 1), which enhances the relaxivity level. Relaxivity calculation is a highly time-consuming procedure, which requires relaxation time measurement in relation to probe concentration. Furthermore, the essential studies of relaxivity dependence per magnetic field are rare and cannot be routinely performed for the design of new contrast agents in a laboratory with a single high-field NMR magnet [103]. Moreover, the relaxation mechanism is non-linearly dependent on the magnetic field strength, which results in nonlinear relaxivity curves per field and requires a high accuracy of experiments. In this work, we present the relaxivities *r*_1_ and *r*_2_ of the HSA-NIT conjugates in PBS measured at 25 °C on two NMR spectrometers (1.88 T and 7 T) and two MRI scanners (3 T and 14.1 T) (Figure 7, Tables S4 and S5). The relaxivity *r*_1_ of HSA-NIT increases from 1.88 T to 3 T with a further decrease per field increase (Figure 7, on the left). A similar behavior of *r*_1_ dependence per field was observed for Gd-based CAs [103,104]. Relaxivity *r*_2_ almost linearly increases from 1.88T to 7 T. The same *r_2_* curve behavior was obtained for ferrous oxide [105] and gadolinium CAs [106]. The increase in the magnetic field to 14.1 T resulted in dramatic drops of *r*_2_. The explanation of this phenomenon requires additional experiments due to the lack of information about contrast agent studies on modern high-field MRI scanners. The HSA-Et/Et-mal conjugate showed the highest relaxivities *r*_1_ and *r*_2_ per molecule of probe (Figure 7). Moreover, HSA-Et/Et-mal showed the highest relaxivities per nitroxide residue among HSA-NIT conjugates (Table S5). The tetramethyl-substituted nitroxide conjugates showed a slightly lower relaxivity *r*_1_ per nitroxide and a 1.5-fold lower *r*_2_ than those of tetraethyl-substituted nitroxides (Table S5).

For commercial Gd-based CAs such as Magnevist, Prohance, Omniscan, Dotarem, etc., in human plasma at 37 °C and 0.47–3 T range, relaxivities *r*_1_ and *r*_2_ do not exceed 4–6 mM^−1^ s^−1^ [10]. HSA-Et/Et-mal exceed the relaxivities of the well-known *T*_1_-CA paramagnetic Gd^3+^ complex (Gadovist) by 2-fold and 14-fold, respectively (*r_1_* = 4.7 mM^−1^s^−1^ and *r_2_* = 6.8 mM^−1^s^−1^ at 1.5 T) [107]. For Gd CAs, the *r*_2_*/r*_1_ ratio is ~1 at 0.5–3 T [10]. In the body, water T_2_ is 5−15 times shorter than T_1_. In this way, Gd-based complexes are utilized in clinics only as CAs for T_1_-weighted images. For HSA-Et/Et-mal, the *r*_2_*/r*_1_ ratio is 2.9 and 3.3 at 1.88 T and 3 T, respectively (Table S4). It seems that at low field HSA-NIT, conjugates can be used as *T*_1_- and *T*_2_-weighted CAs for standard 1.5 T and 3 T MRI scanners. For HSA-NIT, the *r*_2_*/r*_1_ ratio significantly increases with magnetic field, indicating the dominance of *T*_2_ effects and perspectives for *T*_2_-weighted MRI phantom images at high field.

### 2.7. MRI Phantoms of HSA-NIT at 3 T and 14.1 T

**Figure 8:**
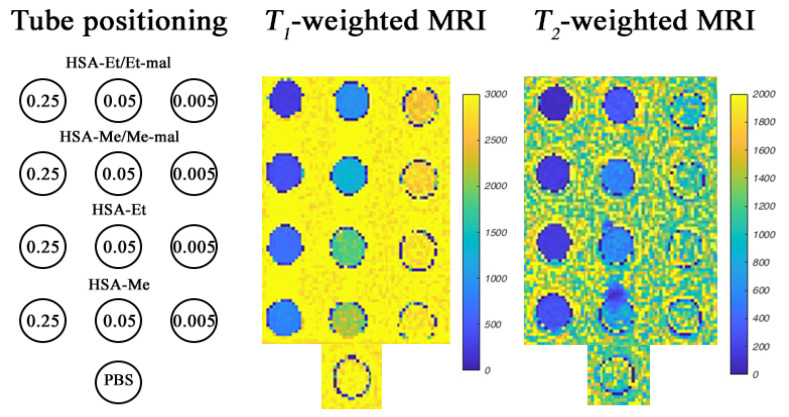
*T*_1_- and *T*_2_-weighted MRI images of HSA-NIT containing phantoms at 25 °C in a 3 T MRI scanner in three HSA-NIT concentrations (0.25 mM, 0.05 mM, and 0.005 mM). MRI images of phantoms at various HSA-NIT concentrations (0.25–0.005 mM per HSA and 0.5–0.05 mM per nitroxide) are presented in Figure S9.

To confirm the possibility of MRI signal registration, phantom images of conjugate solutions were obtained in vitro at protein concentrations from 0.5 mM to 0.005 mM and nitroxide concentration from 0.5 to 0.05 mM in PBS at 3 T in a clinical Siemens 3 T whole body system (Figure 8 and Figure S9).

This method of studying the properties of CAs is widely used due to the ability to perform image registration without the use of living tissues [108]. The presence of contrast efficiency in HSA-NIT for *T*_1_- and *T*_2_-weighted MRI images was determined by comparison with the control PBS solution (Figure 8 at the bottom). As shown in Figure 8 and Figure S9, all HSA-NIT conjugates exhibit dose-dependent signal enhancements, resulting in brighter images as the CA concentrations increase.

The *r*_2_/*r*_1_ of 2.6–4.5 obtained at 3 T suggests that HSA-NIT can be used as *T_1_*- and *T_2_*-CA. The best sensitivity in the registration of phantom images was found for the HSA-Et/Et-Mal sample, which has the highest relaxivity among all conjugates. However, HSA-Me/Me-mal and HSA-Et/Et-mal conjugates were shown to have comparable contrast efficiencies in registering *T*_1_- and *T*_2_-weighted MRI images. MRI phantoms at 14.1 T were measured using a Magnex (Oxford) ultra-high-field MRI system at 25 °C. Good contrast was obtained for *T*_1_-weighted MRI (Figure S10). The best results were provided by HSA-Me/Me-mal and HSA-Et/Et-mal conjugates. *T*_2_-weighted images showed a similarly good contrast for all HSA-NIT.

The MRI phantoms of HSA-NITs provide the possibility of excellent contrast because the conjugates showed good relaxivity, and indicate scope for substantial performance increases for this novel class of ORCAs. The high solubility, stability, and biocompatibility of HSA-NIT also demonstrate the advantages of these ORCAs. Furthermore, such properties of albumin as accumulation in tumors via the Enhanced Permeability and Retention effect and receptor interaction, low immunogenicity, and long lifetime in the bloodstream make HSA-NIT promising for cancer MRI.

### 2.8. Trypsinolysis of HSA-NIT Conjugates and OMRI Application

**Figure 9:**
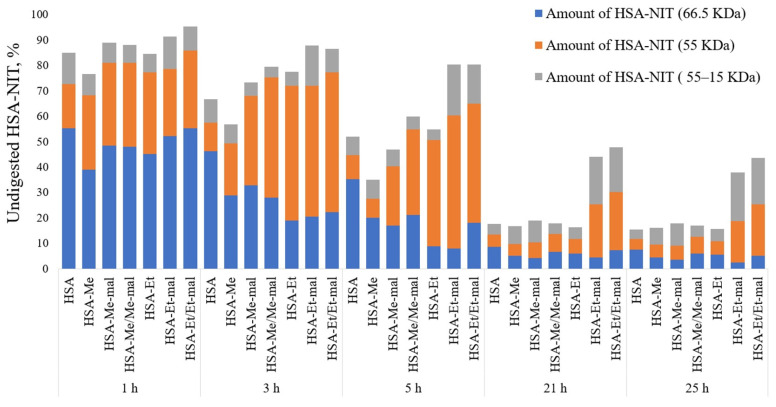
Susceptibility of HSA or HSA-NIT to trypsinolysis analyzed by SDS-PAGE (Figure S11).

HSA is finally catabolized inside various organs [109], and it is preferentially internalized by tumor cells [110]. The proteolysis of HSA was investigated in PBS (pH 7.4) using the enzyme trypsin, and analyzed by SDS-PAGE (Figure 9 and Figure S11). It could be a concern that, due to excessive modification, the trypsinolysis process could be inactive. As shown in Figure 9, none of the HSA-NIT exhibits any strong inhibition of proteolysis when exposed to trypsin, compared to native HSA.

HSA-NIT conjugates faster than native HSA digested to 15–55 kDa fragments, which correlates with previously published data on N-homocysteinylated HSA conjugates [111]. HSA-NIT was specifically sequentially degraded first to a fragment with an MW of ~55 kDa with greater efficiency than subsequent degradation to smaller fragments with MWs from 45 to 15 kDa. The trypsinolysis of the albumin monomer (66 kDa) into the 55 kDa band is highly efficient for all HSA-NIT conjugates. After 24 h, less than 20% of the protein was undigested, except for two samples (HSA-Et/Et-mal and HSA-Et-mal). This effect may be caused by the blocking of an important cleavage site in the 55 kDa fragment upon protein modification in these two conjugates, leading to a slowdown of the proteolysis at this stage.

**Figure 10:**
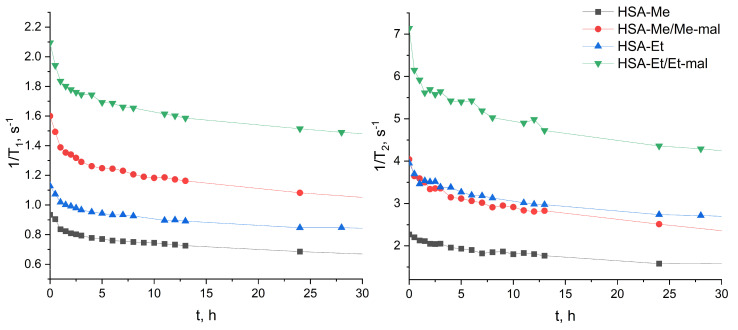
T_1_ and T_2_ relaxation time changes for trypsinolysis of HSA-NIT per time.

Enzymes play vital roles in many essential cellular and tissue processes. The over-expression of enzymes is usually correlated with many pathological conditions and diseases [112]. For example, high proteolytic activities of extracellular and intracellular proteases are observed during tumor growth, invasion, and metastasis [113,114]. Many proteinases are secreted in the cell lysosomal compartment and the extracellular matrix. We hypothesized that HSA-NIT conjugates may accumulate in the tumor by passive transport or due to albumin-binding proteins and receptors [115], degrade by proteases, and release NIT-containing peptides. Upon proteolysis, the MW of NIT-species becomes lower, which leads to a decrease in the correlation time and EPR spectrum line width [32,49,50]. HSA-NIT may serve as an enzyme-activated probe for the in vivo imaging of biological events, and a probe for proteolytic activity. This enzyme-participated cleavage of HSA-NIT would lead to relaxivity *r*_1_ and *r*_2_ changes and an ODNP activation once the small radicals are free in solution, enabling OMRI. To investigate relaxivity changes under proteolysis, we added trypsin to HSA-NIT at 37 °C and measured *T*_1_ and *T*_2_ relaxation times per reaction time. The same reaction condition was used as for the previous trypsinolysis experiment (Figure 9, Section 3.8) except that for NMR, a volume of 500 µL of the reaction mixture was used. The values of *T_1_* and *T_2_* increased significantly for the first 3–4 h and reached a plateau after 12 h of degradation by trypsin (Figure 10). Similarly, the observation of the enhancement in relaxation time during digestion was previously made for protein–nitroxide complexes [32,49]. This phenomenon can be exploited to extend the ability of MRI to follow biological processes associated with the cellular uptake and digestion of proteins [32,49].

We decided to calculate the possible relaxation time of the system, based on the assumption that all nitroxides act as a free radical in solution. Nitroxide relaxivity values of 0.2 (*r*_1_, Me), 0.26 (*r*_2_, Me), 0.24 (*r*_1_, Et), and 0.29 (*r*_2_, Et) mM^−1^s^−1^ at 1.88 T in aqueous solution were measured and used for calculation. Excellent correlations between 1/*T*_1_ values after 24 h and the same number of free radicals were obtained for each sample (Table S6). A high correlation between the plateau values of relaxation time *T*_1_ and the calculated values indicates the great mobility of nitroxides in proteolysis products, which may facilitate good ODNP enhancement. The 1/*T*_2_ values in digested peptides were usually twice as high as those for nitroxides in solution (Table S6).

**Figure 11:**
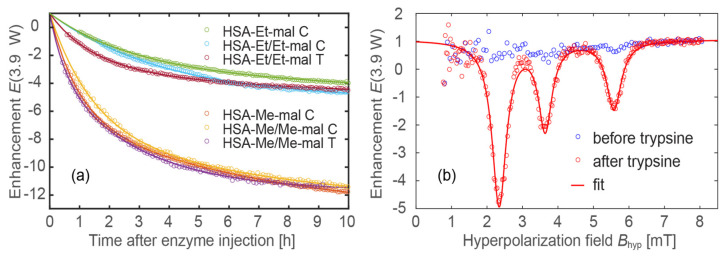
(**a**) Real-time measurement of ODNP enhancement during proteolysis. Circles represent measurement points and lines the fitted exponential curves (Equation (1); for fitting parameters see Table 3). (**b**) ODNP spectra of HSA-Et/Et-mal T before and after the cleavage process (~20 h after injection of trypsin). The sample name has been extended to include the type of protease, either C or T.

To simulate this feature in vitro, we added the proteases, chymotrypsin (C) and trypsin (T), to an HSA-NIT aqueous samples at 37 °C. Trypsin is a serine protease that specifically cleaves at the carboxylic side of lysine and arginine residues. Chymotrypsin hydrolyzes the peptide bonds after aromatic amino acids such as tryptophan, leucine, tyrosine, and phenylalanine. The association of proteolytic enzymes with cancer is well established, with different proteolytic families, including serine proteases [116]. Therefore, chymotrypsin and trypsin enzymes were used as commercially available proteases for the model ODNP experiment. The proteases released the small-molecular-weight nitroxide-based radical from HSA-NIT. The cleavage process was tracked in real time by measuring the ODNP enhancement factor for four samples, two of which were tested with C and T (Table 3, Figure 11a). To properly fit the measured enhancement factor *E*(*t*) as a function of time after protease injection, a double exponential function was used for most samples (except for sample HSA-Et/Et-mal C):

(1) Et=A11−e−tτ1+A21−e−tτ2

The accuracy of this method was estimated to be within ~10% (Figure S12).

This indicates that the cleavage process is dominated by a slow (τ_1_) and a fast (τ_2_) reaction process. This could explain why the nitroxides are cleaved from the HSA at different positions, resulting in the two different time constants. After 20 h, the cleavage process almost stopped for all samples. An ODNP spectrum before and after the cleavage process is shown in Figure 11b, demonstrating the activation of the spin probes. Two samples were tested for C and T (HSA-Me/Me-mal and HSA-Et/Et-mal), but no significant difference in the kinetics was observed (Table 3 and Figure 11a). To check the ODNP efficacy of the cleaved spin probes, a characterization similar to that used in Ref. [46] was performed (Table 3 and Table S7). For ODNP, the most important parameters are the maximum possible enhancement factor, *E*_max_, and *P*_1/2_, the RF power needed to reach *E*_max_/2. Obviously, it would be best to have a high enhancement factor at a low RF power level for future in vivo experiments, since it is mandatory not to heat tissue; therefore, the specific absorption rate of the HF irradiation should be kept as small as possible. As an example, TEMPO free radicals in a buffer solution have an *E*_max_ = 153.7 and *P*_1/2_ = 6.2 W.

Although the here-presented spin probes after the cleavage processes do not show an equivalently good performance compared to TEMPO free radicals, there are many possibilities for improvements, e.g., the ^15^N labeling or deuteration of the used spin probes. Nevertheless, the *E*_max_ enhancement of 40–50 with a low *P*_1/2_ of 10–12 W grounds the high potential applicability of HSA-NIT conjugates for OMRI. Note that *P*_1/2_ is different for tetramethyl and tetraethyl substituted nitroxides. The tetramethyl-substituted nitroxides exhibit a *P*_1/2_ range of 10–12 W, while the tetraethyl-substituted nitroxides exhibit a *P*_1/2_ range of 20–25 W. This is consistent with the findings of reference [46], where the tetraethyl-substituted nitroxides also exhibited a greater *P*_1/2_ compared to the tetramethyl-substituted nitroxides. In all experiments shown in Figure 11, the same RF power, *P* = 3.9 W, was used, so the enhancement *E*(*P* = 3.9 W) of the tetraethyl-substituted nitroxides was lower than that of the tetramethyl-substituted nitroxides.

The possible enzymatic hydrolysis and ODNP enhancement make HSA-NIT stimuli-responsive, activatable OMRI HCAs for use in cancer imaging [49,51,117]. Protein digestion switches from a stable, low-signal state to one with a high signal (Figure 11b). HSA-NIT is one of the rare examples of nitroxide-based OMRI systems with high enhancement and low power, which is soluble in aqueous solution, biocompatible, and prospective for use in cancer tissue delivery. However, the sluggish nature of the protease cleavage process (>1 h) requires further investigation. If the decay/clearance rate of the released nitroxides exceeds the duration of the cleavage process, no ODNP effect can be observed. Therefore, the stability of nitroxides is of utmost importance for in vivo experiments.

## 3. Materials and Methods

### 3.1. Chemicals

Human serum albumin (HSA) was purchased from Sigma-Aldrich (cat. no. A3782, St. Louis, MO, USA). Ellman’s test indicated that the albumin preparation contained 0.30 ± 0.06 thiol groups per molecule. MS (MALDI ToF) m/z HSA 66.48 kDa. Nitroxyl radical derivatives 3-carboxy-2,2,5,5-tetramethyl-4-[(2-oxothiolan-3-yl)carbamoyl]-2,5-dihydro-1*H*-pyrrol-1-oxyl (Me) [23], 3-carboxy-2,2,5,5-tetraethyl-4-[(2-oxothiolan-3-yl)carbamoyl]-2,5-dihydro-1*H*-pyrrol-1-oxyl (Et) [23], 3-maleimido-2,2,5,5-tetramethylpyrrolidine-1-oxyl (Me-mal) [118], and 3-maleimidomethyl-2,2,5,5-tetraethylpyrrolidine-1-oxyl (Et-mal) [119] were prepared and kindly provided by LAS NIOH SB RAS (Novosibirsk, Russia). 5,5′-dithio-bis(2-nitrobenzoic acid) (DTNB), all solvents, and other reagents were purchased from Sigma (St. Louis, MO, USA) at the highest available grade and used without purification. MTT (3-[4,5-dimethylthiazol-2-yl]-2,5-diphenyltetrazolium bromide) was purchased from Invitrogen (Waltham, MA, USA). Centricon concentrators with a 3 kDa molecular weight cut-off were purchased from Millipore. Trypsin for protein trypsinolysis was obtained from Gibco (cat. no. 15090046, Carlsbad, CA, USA). All other chemicals were purchased from Sigma-Aldrich at the highest available grade and used without purification. Aqueous solutions were prepared using bidistilled water, and protein solutions were prepared using phosphate-buffered saline (PBS) (15 mM KH_2_PO_4_, 145 mM NaCl, pH = 7.4).

### 3.2. Methods

Electronic absorption spectra were recorded on a UV-1800 spectrometer (Shimadzu, Tokyo, Japan). The concentrations of HSA solutions in PBS (pH 7.4) were determined by absorption at 278 nm, using the molar extinction coefficient ε = 3.7 × 10^4^ M^−1^cm^−1^ [23].

The number of thiol groups per HSA molecule was determined using Ellman’s method with DTNB [120]. A solution of HSA (200 μL, 0.1 mM) in 0.0074 M phosphate buffer (pH = 8.1) was mixed with DTNB (53.3 μL, 0.01 M) in 0.037 M phosphate buffer (pH = 8.0) and EDTA (13 μL, 0.025 M) in 0.0074 M phosphate buffer (pH = 8.1) and incubated for 40 min at room temperature. Unmodified HSA as a control was prepared using 200 μL of 0.0074 M phosphate buffer (pH = 8.1). The concentration of the yellow-colored product was measured using a UV-1800 spectrometer at 412 nm (ε_412_ = 1.42∙10^4^ M^−1^cm^−1^) relative to the comparison solution, and the concentration of thiol groups was calculated.

HSA conjugates were analyzed by 7% and 10% sodium dodecyl sulfate-polyacrylamide gel electrophoresis (SDS-PAGE) under Laemmli conditions with subsequent Coomassie Brilliant Blue staining. For native PAGE, SDS was excluded from the loading buffer and gel solutions. Quantitative data were obtained by digitizing the gel using GelPro Analyzer software 3.0 (Media Cybernetics, Rockville, MD, USA).

Circular dichroism (CD) data were collected for HSA conjugates (50 μM, 100 μL) in PBS at 25 °C with a JASCO J-600 spectrophotometer with a time constant of 4 s and a bandwidth of 1 nm, using a 0.01 cm path length quartz cell. All CD spectra were obtained from 190 to 240 nm, and the final spectrum was obtained as an average of 20 spectra. The obtained CD spectra were processed by extrapolation from the basis spectra of α-helices, β-sheets, and the disordered structures taken from the CCA+ software 32bit 0.2b1. The percentages of secondary structures in HSA were calculated from the obtained data [94].

Matrix-assisted laser desorption/ionization time-of-flight mass spectrometry (MALDI-ToF MS) of HSA conjugates were recorded on a Bruker Autoflex Speed (Bruker, Germany) MALDI-ToF mass spectrometer in a positive linear mode. A smart beam-II laser was used with 2,5-dihydroxyacetophenone (2,5-DHAP) as the matrix. Protein samples were desalted by ZipTip C4 pipette tips. A 2 µL protein sample solution was mixed with 2 µL of 2% TFA (trifluoroacetic acid). For the latter solution, 2 µL of the matrix (2,5-DHAP) was added. The mixture was pipetted up and down until the crystallization started. Approximately 1 µL of the resulting solution was deposited on the 384-grit steel target plate and allowed to dry before being introduced into the mass spectrometer. Mass spectra were obtained by averaging 2500–3000 laser shots. MALDI-ToF data were deconvoluted using the software package mMass 5.5.0 [121,122,123].

### 3.3. Electron Paramagnetic Resonance (EPR)

Samples were placed in quartz capillary tubes (OD 1.5 mm, ID 0.9 mm). Continuous wave (CW) EPR measurements were performed in the X-band at 25 °C using an X-band Bruker EMX spectrometer. The experimental continuous wave EPR settings were as follows: central field 350.98 mT, sweep width 12 mT, microwave power 6.331 mW, microwave frequency 9.59 GHz, modulation frequency 100 kHz, modulation amplitude 0.15 mT, conversion time 81.92 ms, number of points 1024, and number of scans 10. Double integrals were calculated using the Bruker WinEPR software 4.3. The radical concentration was determined by comparing the spectral double integrals of the sample and a TEMPO ((2,2,6,6-tetramethylpiperidin-1-yl)oxyl) nitroxide solution with a known concentration. The experimental spectra were simulated using EasySpin software 5.2.35 [124,125]. All spectra were simulated in the slow-motion regime with the EasySpin chili function. Experimental EPR spectra were decomposed using EasySpin software 5.2.35 into three components with different rotational correlation times—“slow”, “fast” and “free”. The rotational correlation time shows the mobility of the label: longer times correspond to more immobilized labels and vice versa. The g-tensor and hyperfine tensor A values were the same in all spectra: g_xx_ = 2.009, g_yy_ = 2.006, g_zz_ = 2.0025, A_xx_ = A_yy_ = 0.5; the values of A_zz_ were varied. Line broadenings were calculated assuming Voigtian line shape—the convolution of Lorentzian and Gaussian line shape.

### 3.4. Fractionation of HSA by Gel-Filtration Chromatography

The monomer HSA fraction was obtained by gel-filtration chromatography using a Sephadex-G150 superfine according to the previously published procedure [59,126]. The parameters of the chromatography column are 30 cm height, 2 cm diameter, and 5 mm outlet diameter. The Sephadex G-150 was suspended in 0.1 M KCl according to the standard manufacturing procedure. Then, 1 mL of a 1 mM HSA solution in 0.1 M KCl was applied to the Sephadex column. The solution of 0.1 M KCl was then passed through the column. The absorbance of the eluate was monitored with a UV- 1800 spectrometer (Shimadzu, Japan). Each eluate probe was analyzed by SDS-PAGE. After that, HSA fractions were combined and concentrated using Centricon Amicon Ultra 3K concentrators, and PBS buffer was used as an eluent.

### 3.5. HSA-NIT Conjugates Synthesis Methods

#### 3.5.1. Method 1: HSA-NIT Synthesis Method with Sequential Addition of Nitroxide Derivatives

To 0.15 mL of a 0.5 mM HSA monomer in PBS, a 30-fold molar excess of HTL-NIT dissolved in 7.5 μL DMSO was added. Synthesis was carried out at 37 °C for 26 h under 250 rpm stirring. The reaction mixture was centrifuged at 10,000× *g* for 15 min to remove the poorly soluble nitroxide excess. The HSA-NIT conjugate was cleaned using Centricon concentrators (3 kDa molecular weight cutoff; Amicon Centriprep YM30, Millipore, Bedford, MA) according to the procedure described by the manufacturer, with PBS as the eluent. The amount of nitroxide label was analyzed by CW EPR. The HSA-Me yield was 95% with a modification degree of 4.8 ± 0.4. The HSA-Et yield was 88%, with a modification degree of 5.2 ± 0.5. According to SDS-PAGE data (Figure 1), the protein oligomer contents in HSA-Me and HSA-Et are 12.8 ± 1.4% and 11.1 ± 1.5%, respectively.

To 0.15 mL of 0.5 mM synthesized HSA-Me and HSA-Et in PBS, 15-fold molar excesses of Me-mal and Et-mal, respectively, dissolved in 7.5 μL DMSO were added. Synthesis was carried out at 37 °C for 26 h under 250 rpm stirring with the same cleaning procedures. The amount of nitroxide label was analyzed by CW EPR. The HSA-Me/Me-mal yield was 94% with a modification degree of 7.0 ± 0.6. The HSA-Et/Et-mal yield was 86% with a modification degree of 10.2 ± 0.8. According to SDS-PAGE data (Figure 1), the protein oligomer contents in HSA-Me/Me-mal and HSA-Et/Et-mal are 20.2 ± 1.5% and 17.3 ± 1.7%, respectively.

#### 3.5.2. Method 2: HSA-NIT Synthesis Method with Simultaneous Addition of Nitroxide Derivatives

To 0.1–0.3 mL of a 0.5, 0.1, or 0.05-mM HSA monomer in PBS, a 30–900-fold molar excess of HTL-NIT and a 15–450-fold molar excess of mal-NIT dissolved in DMSO were added (Table S1). The resulting DMSO amount in the solution was in the 5–10% range. Synthesis was carried out at 37 °C for 22–64 h under 250 rpm stirring. The reaction mixture was centrifuged at 10,000× *g* for 15 min to remove poorly soluble nitroxide excess. The HSA-NIT conjugate was cleaned using Centricon concentrators (3 kDa molecular weight cutoff; Amicon Centriprep YM30, Millipore, Bedford, MA) according to the procedure described by the manufacturer, with PBS as the eluent. The amount of nitroxide label was analyzed by CW EPR. The yield for HSA-NIT conjugates is in the 85–90% range (Table S1).

#### 3.5.3. Method 3: HSA-NIT Synthesis Method with Simultaneous Addition of Nitroxide Derivatives Aliquots in Equal Time Intervals

To 0.64 mL of a 0.274 mM HSA monomer in PBS, 50 μL DMSO was added and stirred at 450 rpm for 5 min. To the resulting mixture, 80-fold molar excesses of HTL-NIT and mal-NIT in DMSO were added in aliquots after time intervals set according to Table S2. For comparison, reactions with only HTL-NIT or mal-NIT were carried out. Synthesis was carried out at 37 °C for 96 h under 250 rpm stirring. The reaction mixture was centrifuged at 10,000× *g* for 15 min to remove poorly soluble nitroxide excess. The HSA-NIT conjugate was cleaned using Centricon concentrators (3 kDa molecular weight cutoff; Amicon Centriprep YM30, Millipore, Bedford, MA) according to the procedure described by the manufacturer, with PBS as the eluent. The amount of nitroxide label was analyzed by CW EPR and MALDI-ToF. The molecular weight of the HSA monomer used in the experiment according to MALDI-TOF was 66.48 kDa.

HSA-Me. The yield was ~94%. MALDI TOF MS m/z 70.585 kDa (~14.4 ± 0.6 NIT residues). The average modification degree calculated by MALDI-TOF MS and CW EPR is 13.7 ± 0.7. HSA-Me/Me-mal. The yield was ~85%. MALDI TOF MS m/z 71.198 kDa (~18.1 ± 0.8 NIT residues). The average modification degree calculated by MALDI-TOF MS and CW EPR is 17.1 ± 1.0. HSA-Me-mal. The yield was ~93%. The modification degree calculated by CW EPR is 16.4 ± 0.9. 

HSA-Et. The yield was ~92%. MALDI TOF MS m/z 71.014 kDa (~13.2 ± 0.3 NIT residues). The average modification degree calculated by MALDI-TOF MS and CW EPR is 13.1 ± 0.2. HSA-Et/Et-mal. The yield was ~84%. MALDI TOF MS m/z 73.246 kDa (~20.9 ± 0.8 NIT residues). The average modification degree calculated by MALDI-TOF MS and CW EPR is 21.9 ± 1.0. HSA-Et-mal. The yield was ~90%. The modification degree calculated by CW EPR is 20.8 ± 1.1.

### 3.6. Relaxivities (r_1_ and r_2_) of HSA-NIT Conjugates

Relaxivities of the HSA-NIT were measured at 25 °C and 80 MHz (1.88 T), 128 MHz (3.0 T), 300 MHz (7.0 T), and 600 MHz (14.1 T) in aqueous PBS buffer (pH 7.4) using a Magritek Spinsolve80 spectrometer, a clinical Siemens 3T whole body system, a Bruker Avance III 300 MHz spectrometer, and a Magnex (Oxford) ultra-high-field MRI system, respectively. Inversion recovery (*T*_1_) and Car–Purcell–Meiboom–Gill (*T*_2_) pulse sequences were used. The relaxivities, *r*_1_ and *r*_2_, were calculated according to Equation (2) for HSA-NIT in PBS. All reported relaxivity values were the average of three or more independent measurements.

(2) $$\frac{1}{T_{i}}=\frac{1}{T_{i,d}}+r_{i}\cdot C_{HSA-NIT},$$

where *T_i_* is the measured *T*_1_ (*i* = 1) or *T*_2_ (*i* = 2), *T_i,d_* is the diamagnetic contribution of the solvent and C_HSA_-_NIT_ is the conjugate concentration.

### 3.7. Reduction of HSA-NIT Conjugates

The reduction rate constants of HSA conjugates were investigated using two methods: EPR and *T*_1_-relaxation time measuring [23]. For a typical experiment, HSA-NIT (0.09 mM), ascorbic acid (39 mM), and glutathione (19.5 mM) concentrations in the reaction mixture were used.

#### 3.7.1. Reduction Studies by T_1_-Relaxation Time Measuring

The NMR tube with a solution of HSA-NIT was mixed with Asc/GSH in PBS buffer and immediately placed in the Magritek Spinsolve80 NMR spectrometer, and the 1/*T*_1_-relaxation rates were measured and calculated using the program MestReNova. The reduction constants were calculated as a pseudo-first-order rate from the dependence of the logarithm of the difference of the relaxation rates (1/*T*_1_ and 1/*T*_1,*d*_) versus time, divided by the concentration of ascorbic acid (Equation (3)).

(3) $$\ln\left(\frac{1}{T_{1}}-\frac{1}{T_{1,d}}\right)=\ln\left(r_{1}\cdot C_{NIT}^{0}\right)-k\cdot C_{Asc}\cdot t$$

#### 3.7.2. Reduction Studies by EPR

The solution of HSA-NIT with Asc/GSH (10 µL) in PBS buffer was drawn into a capillary tube, sealed from both sides, and placed into the resonator of the spectrometer. The double integral of the spectrum was measured as a function of time. The reduction constant was calculated as a pseudo-first-order rate equation.

### 3.8. Trypsinolysis of HSA-NIT Conjugates

The solution of HSA-NIT 150 μL of 0.2 mM in PBS buffer (pH 7.4) was digested with 6 μL 0.1 mM trypsin («Gibco» cat. No. 15090046) at an enzyme–substrate ratio of 1:50 at 37 °C and 250 rpm on a shaker for 25 h, with aliquots taken after 1, 3, 5, 21 and 25 h. The latter solution aliquot (7.5 µL) was mixed 1:1 with SDS-PAGE sample buffer (50 mM tris hydrochloride pH 6.8, 1% SDS, 10% glycerol, and 0.025% bromophenol blue with or without 0.1% DTT), denatured for 5 min at 95 °C, and subjected to SDS-PAGE. Albumin bands were visualized by Coomassie blue staining. Quantitative data were obtained by digitizing the gel using GelPro Analyzer software 3.0 (Media Cybernetics). Bands in the gel with a molecular weight of 66.5 kDa (HSA monomer) were labeled as undigested albumin.

### 3.9. The Acquisition of OMRI Spectra of HSA-NIT Conjugates

**Figure 12:**
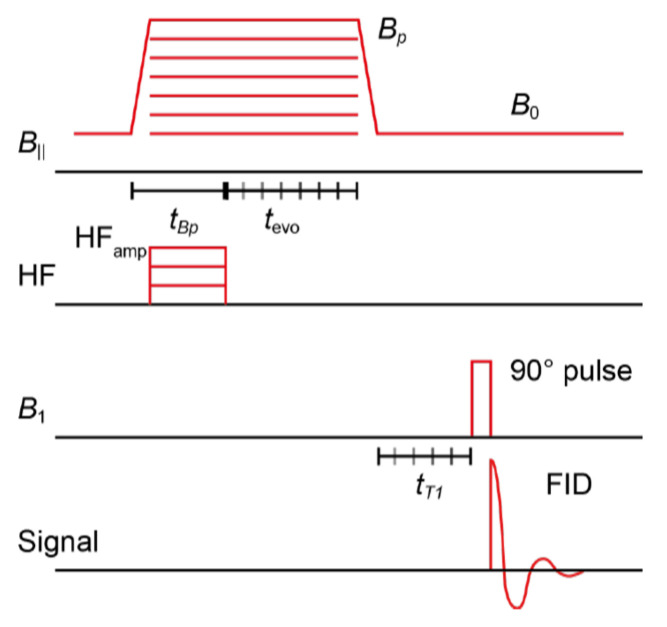
Schematic of the used sequences for the ODNP efficacy characterization.

The experimental measurements were performed using a simple free induction decay (FID) sequence following a hyperpolarization phase, as shown in Figure 12.

During the hyperpolarization phase, a magnetic field of magnitude *B_p_* was applied orthogonally to the *B*_0_ field (~94 µT), accompanied by an RF pulse of duration *t_Bp_* and frequency on the scale of the electron Larmor frequency. The FID readout procedure consisted of a 90-degree pulse followed by data acquisition. The acquisition of OMRI spectra involved the use of a radio frequency (RF) pulse with a constant amplitude and frequency (~120 MHz) while varying the magnitude of the magnetic field, *B_p_*, for each data point. The time domain data collected during the acquisition phase were then Fourier-transformed. The resulting integrated MR peak was then plotted as a function of *B_p_* amplitude. To measure the hyperpolarization buildup rate, the length *t_Bp_* of the hyperpolarization phase is varied. By fitting an exponential buildup function to the integrated MR peak as a function of *t_Bp_*, the hyperpolarization buildup rate *T*_Hyp_ can be determined. Note that *T*_Hyp_ cannot be experimentally distinguished from the longitudinal relaxation time *T*_1_(*B_p_*).

The longitudinal relaxation time *T*_1_ was measured at two different field strengths, *B_p_* and *B*_0_. To measure *T*_1_(*B_p_*), the additional delay *t*_evo_ is varied after the RF field is turned off and before the ramp-down of *B_p_*. For the measurement of *T*_1_(*B*_0_), an additional delay *t_T_*_1_ is inserted after the ramp-down of *B_p_* and before the readout phase is inserted. An exponential decay function was fitted to the integrated MR peak as a function of the delays *t*_evo_ and *t_T_*_1_. As a result, *T*_1_(*B_p_*) and *T*_1_(*B*_0_) were determined from the fit.

An expression for the power-dependent enhancement factor is given by Equation (4).

(4) $$E\left(P\right)=\frac{\left(E_{max}-1\right)P}{P_{\frac{1}{2}}+P}+1$$

By varying the RF power *P* and plotting the integrated MR peak as a function of *P*, the above dependence can be fitted, and *E*_max_ and *P*_1/2_ determined. Note that *P*_1/2_ is a setup-dependent parameter. A systematic study was performed to compare this parameter with those in other studies. OMRI applications require spin probes with a high *E*_max_ and low *P*_1/2_.

### 3.10. Cytotoxicity Assay

Cytotoxicity studies were performed by colorimetric assay using the MTT compound on human breast adenocarcinoma (MCF-7) and human glioblastoma (T98G) tumor cell lines. MCF-7 and T98G cells were cultured in IMDM or EMEM medium, respectively, supplemented with 10% fetal bovine serum (FBS) (Invitrogen), penicillin (100 units/mL) and streptomycin (100 units/mL) at 37 °C and 5% CO_2_ in a humidified atmosphere. Exponentially growing cells were plated in a 96-well plate (2000 cells per well). After overnight incubation, the cells were treated with media containing HSA-NIT conjugates. The solutions of conjugates were applied to the media for 72 h at 37 °C. An aliquot of MTT solution (10 μL, 25 mg/mL) in PBS was added to each well and incubated at 37 °C for 3 h. The medium was removed and the precipitate was dissolved in 0.1 mL of isopropanol. The absorbance at 570 nm (peak) and 620 nm (baseline) was read using a microplate reader, Multiscan EX (Thermo Electron Corporation). Results have been expressed as a percentage of control survival values obtained for cells without the addition of albumin conjugates. All values in the present study are given as the mean ± standard deviation (SD) values, and all measurements were repeated three times. 

## Figures and Tables

**Table 1:** HSA-NIT conjugate secondary structure.

Sample	NIT Residue Per HSA Molecule	CD Spectral Analysis	
		α-Helix, %	β-Sheet, %
Monomer HSA	-	55.0 ± 1.0	4.8 ± 1.0
HSA-Me	13.7 ± 0.7	49.2 ± 1.5	5.9 ± 0.6
HSA-Me-mal	16.4 ± 0.9	44.3 ± 1.7	8.1 ± 0.6
HSA-Me/Me-mal	17.1 ± 1.0	43.7 ± 2.0	7.8 ± 0.8
HSA-Et	13.1 ± 0.2	48.8 ± 1.4	6.4 ± 0.6
HSA-Et-mal	21.9 ± 1.0	44.1 ± 1.7	10.9 ± 0.7
HSA-Et/Et-mal	20.8 ± 1.1	43.5 ± 2.0	9.7 ± 0.8

**Table 2:** Second-order rate constants *, *k* (M^−1^s^−1^) of HSA-NIT reduction in PBS (pH 7.4) at 25 °C by EPR and *T*_1_ relaxation time measuring.

Sample	Method	k_init_, M^−1^s^−1^	k_“free” NIT_, M^−1^s^−1^
HSA-Me	EPR	0.044 ± 0.006	0.097 [100], 0.063 [98]
	*T*_1_-meas.	0.027 ± 0.005	
HSA-Me-Mal	EPR	0.041 ± 0.006	
HSA-Me/Me-Mal	EPR	0.043 ± 0.008	
	*T*_1_-meas.	0.033 ± 0.006	
HSA-Et	EPR	0.0019 ± 0.0005	0.001 [98], 0.002 [101]
	*T*_1_-meas.	0.0015 ± 0.0001	
HSA-Et-Mal	EPR	0.0016 ± 0.0004	
HSA-Et/Et-Mal	EPR	0.0015 ± 0.0004	
	*T*_1_-meas.	0.0007 ± 0.0001	

* The kinetics of nitroxide reduction in the presence of ascorbate/glutathione are summarized in Figures S7 and S8.

**Table 3:** Characterization of ODNP efficacy and reaction kinetics of HSA-NIT conjugates after proteolysis.

Sample	*A*_1_	*A*_2_	τ_1_ [1/h]	τ_2_ [1/h]	*P*_1/2_ [W]	*|E*_max_|
HSA-Me-mal C *	−7.4 ± 0.1	−7.2 ± 0.1	6.9 ± 0.2	0.9 ± 0.1	10.8 ± 1.2	54 ± 3
HSA-Me/Me-mal C	−5.7 ± 0.3	−8.4 ± 0.3	8.3 ± 0.9	1.4 ± 0.1	10.3 ± 1.2	50 ± 3
HSA-Me/Me-mal T	−8.7 ± 0.4	−4.0 ± 0.5	2.8 ± 0.2	0.5 ± 0.1	10.9 ± 1.1	47 ± 2
HSA-Et-mal C	−2.8 ± 0.1	−3.9 ± 0.3	18.2 ± 7.4	2.7 ± 0.3	22.6 ± 3.9	45 ± 5
HSA-Et/Et-mal C	−6.2 ± 0.1	–	3.9 ± 0.1	–	21.2 ± 4.0	39 ± 5
HSA-Et/Et-mal T	−2.4 ± 0.1	−3.6 ± 0.1	6.9 ± 0.6	1.4 ± 0.1	24.3 ± 3.1	42 ± 3

* The sample name has been extended to include the type of protease, either C or T, for which the reaction kinetics were monitored.

## Data Availability

Data are contained within the article and Supplementary Materials.

## Supplementary Materials

The following supporting information can be downloaded at: https://www.mdpi.com/article/10.3390/ijms25074041/s1.
