# Supplementary material for: Contrast Agents Based on Human Serum Albumin and Nitroxides for 1H-MRI and Overhauser-Enhanced MRI
Source: Int J Mol Sci. 2024 Apr 5;25(7):4041. doi: 10.3390/ijms25074041 (PMC11012161; doi:10.3390/ijms25074041)
Supplement: Supplementary file 1 [file ijms-25-04041-s001.zip › ijms-2914011-supplementary.pdf]

Contrast Agents Based on Human Serum Albumin and Nitroxides for ^{1}H-MRI and Overhauser-enhanced MRI

Dmitry Mitin [1], Friedemann Bullinger [2], Sergey Dobrynin [3], Jörn Engelmann [2], Klaus Scheffler [2,4], Mikhail Kolokolov [5], Olesya Krumkacheva [5], Kai Buckenmaier [2], Igor Kirilyuk [3,*], and Alexey Chubarov [1,*]

1 Institute of Chemical Biology and Fundamental Medicine, SB RAS 630090 Novosibirsk, Russia
2 High-Field Magnetic Resonance Center, Max Planck Institute for Biological Cybernetics; Tübingen, 72076, Germany
3 N.N. Vorozhtsov Institute of Organic Chemistry SB RAS, 630090 Novosibirsk, Russia;
4 Department of Biomedical Magnetic Resonance, Eberhard-Karls University; Tübingen, 72076, Germany
5 International Tomography Center SB RAS, 630090 Novosibirsk, Russia

1. **Synthesis of HSA-NIT conjugates** 2
2. **Characterization of HSA-NIT conjugates using EPR** 3
3. **MALDI-ToF MS spectra of HSA-NIT conjugates** 4
4. **Absorption spectra of HSA-NIT conjugates** 5
5. **PAGE of HSA-NIT conjugates** 5
6. **Reduction of HSA-NIT conjugates** 6
7. **Relaxivity r_1 and r_2 and MRI phantoms of HSA-NIT conjugates** 7
8. **Trypsinolysis of HSA-NIT conjugates** 9
9. **Longitudinal relaxation times at ULF** 10
10. **Reproducibility of real-time trypsin-mediated cleavage tracking** 10

1. Synthesis of HSA-NIT conjugates

Table S1. Conditions, yields, and modifications for HSA-NIT synthesis method with simultaneous addition of NIT derivatives.

Sample	C_{HSA}, mM	$n_{HTL\text{-}NIT}/n_{HSA}$	$n_{mal\text{-}NIT}/n_{HSA}$	$V_{HTL\text{-}NIT}$, µL	$V_{mal\text{-}NIT}$, µL	ΔV_{DMSO}, µL	V_{Σ}, µL	t_{react}, h	Yield, %	n*
HSA-Me	0.5	30	-	0.75	-	4.25	100	22	95	4.0
		60	-	2.25	-	5.75	150		95	4.7
		180	-	6.75	-	1.25			94	5.5
	0.1	300	-	4.50	-	17.00	300	28	93	5.9
HSA-Me/Me-mal	0.5	30	15	0.75	0.50	4.00	100	22	88	8.1
		60	15	2.25	0.75	5.00	150		88	8.8
		180	45	6.75	2.25	-			87	9.3
	0.1	300	150	2.25	1.50	12.75		41	86	12.6
								64	86	13.7
HSA-Et	0.5	30	-	0.75	-	4.50	100	22	94	4.2
HSA-Et/Et-mal	0.5	30	15	0.75	0.50	4.00	100	22	86	9.9
	0.1	300	150	2.25	1.50	12.75	150	41	85	13.9

n* – The average NIT residues per HSA molecule

Table S2. The addition conditions of NIT in HSA-NIT synthesis method by fractional addition.

NIT addition time	0 h		3 h		24 h		48 h		72 h	
Sample	$V_{HTL\text{-}NIT}$, µL	$V_{Mal\text{-}NIT}$, µL	$V_{HTL\text{-}NIT}$, µL	$V_{Mal\text{-}NIT}$, µL	$V_{HTL\text{-}NIT}$, µL	$V_{Mal\text{-}NIT}$, µL	$V_{HTL\text{-}NIT}$, µL	$V_{Mal\text{-}NIT}$, µL	$V_{HTL\text{-}NIT}$, µL	$V_{Mal\text{-}NIT}$, µL
HSA-Me	2.75	-	-	-	2.75	-	2.75	-	-	-
HSA-Me-mal	-	-	-	3.11	-	3.11	-	3.11	-	-
HSA-Me/Me-mal	2.06	-	-	3.11	2.06	3.11	2.06	3.11	2.06	-
HSA-Et	2.33	-	-	-	2.33	-	2.33	-	-	-
HSA-Et-mal	-	-	-	2.75	-	2.75	-	2.75	-	-
HSA-Et/Et-mal	1.75	-	-	2.75	1.75	2.75	1.75	2.75	1.75	-

2. Characterization of HSA-NIT conjugates using EPR

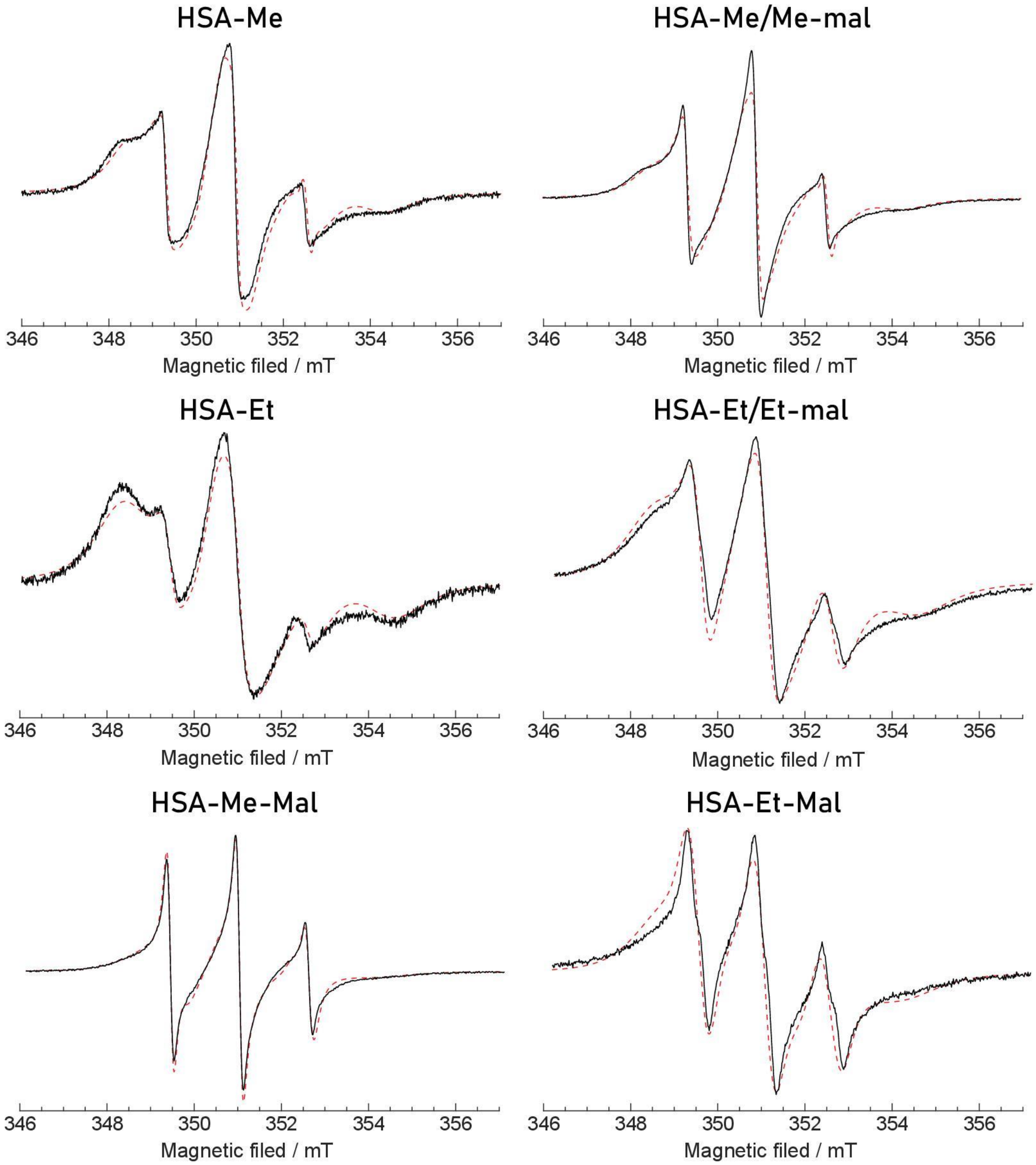

Figure S1. EPR spectra of HSA-NIT conjugates synthesized by method 3.

Spectra were simulated assuming the presence of a set of fractions characterized by different rotational correlation times (τc) and line widths. The g-tensor gii and hyperfine tensor Axx, Ayy values were the same in all spectra: gxx=2.009, gyy=2.006, gzz=2.0025, Axx=Ayy=0.5; the values of Azz were varied. Line broadenings were calculated assuming Voigtian line shape – convolution of Lorentzian and Gaussian line shape.

Table S3. The parameters for EPR simulations in Figure S1.

Sample	Component	τ_c, ns	A_{zz}, mT	Weight, %	Gaussian peak-to-peak line width, mT	Lorentzian peak-to-peak line width, mT
HSA-Me	Slow	8.55	3.41	71%	0.20	0.44
	Fast	1.07	3.82	29%	0.00	0.45
	Free	0.01	3.84	1%	0.19	0.00
HSA-Me-mal	Slow	4.17	3.46	78%	0.46	0.33
	Fast	0.28	3.80	21%	0.02	0.20
	Free	0.04	2.93	1%	0.35	0.01
HSA-Me/Me-mal	Slow	8.13	3.35	66%	0.20	0.49
	Fast	0.93	3.74	33%	0.00	0.40
	Free	0.01	3.84	1%	0.19	0.00
HSA-Et	Slow	7.26	3.61	95%	0.48	0.67
	Fast	0.54	3.73	5%	0.00	0.49
	Free	0.02	3.88	0%	0.25	0.00
HSA-Et-mal	Slow	11.75	2.89	86%	1.19	0.77
	Fast	0.43	3.58	14%	0.46	0.18
	Free	0.02	3.17	0%	0.03	0.07
HSA-Et/Et-mal	Slow	8.55	3.41	83%	0.66	0.87
	Fast	0.61	3.56	17%	0.05	0.61
	Free	0.02	3.52	0%	0.28	0.07

3. MALDI-ToF MS spectra of HSA-NIT conjugates

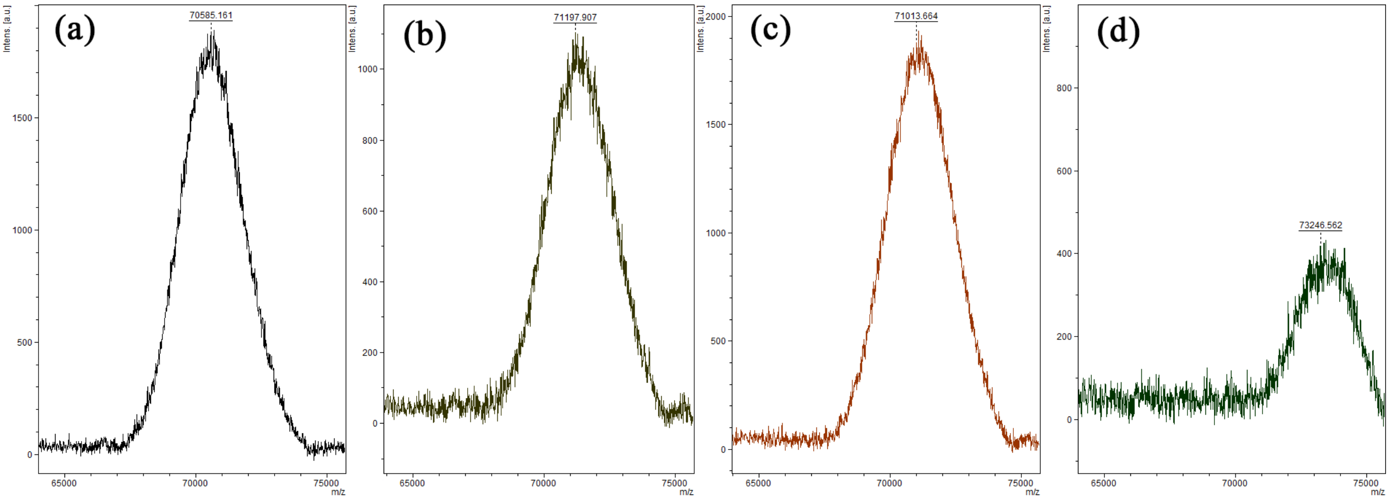

Figure S2. MALDI-ToF MS spectra of HSA-Me (a), HSA-Me/Me-mal (b), HSA-Et (c), and HSA-Et/Et-mal (d) synthesized by method 3.

4. Absorption spectra of HSA-NIT conjugates

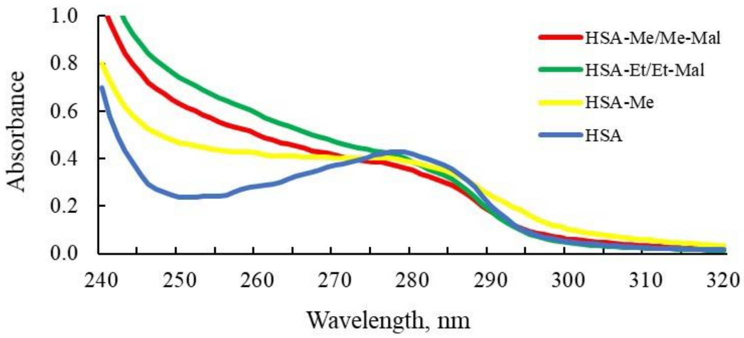

Figure S3. Absorption spectra of HSA-NIT conjugates synthesized by method 3.

5. PAGE of HSA-NIT conjugates

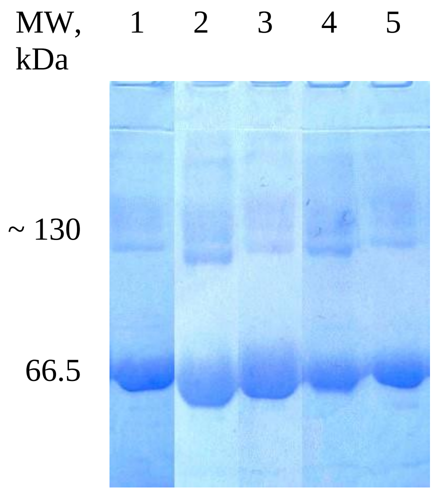

Figure S4. PAGE of HSA-NIT synthesis method 3 under Laemmli condition with the addition of SDS with subsequent Coomassie blue staining. HSA (lane 1); HSA-Me (lane 2); HSA-Me/Me-mal (lane 3); HSA-Et (lane 4); HSA-Et/Et-mal (lane 5).

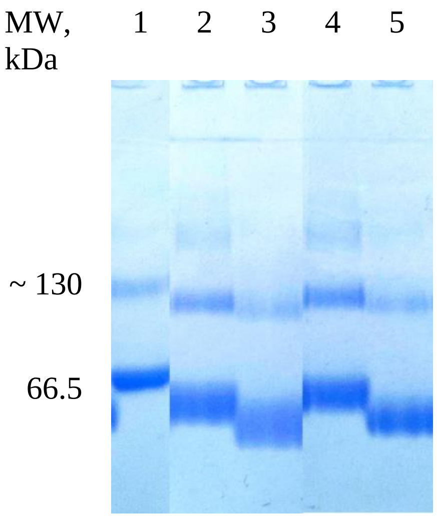

Figure S5. PAGE of HSA-NIT synthesis method 3 under Laemmli condition without addition of SDS with subsequent Coomassie blue staining. HSA (lane 1); HSA-Me (lane 2); HSA-Me/Me-mal (lane 3); HSA-Et (lane 4); HSA-Et/Et-mal (lane 5).

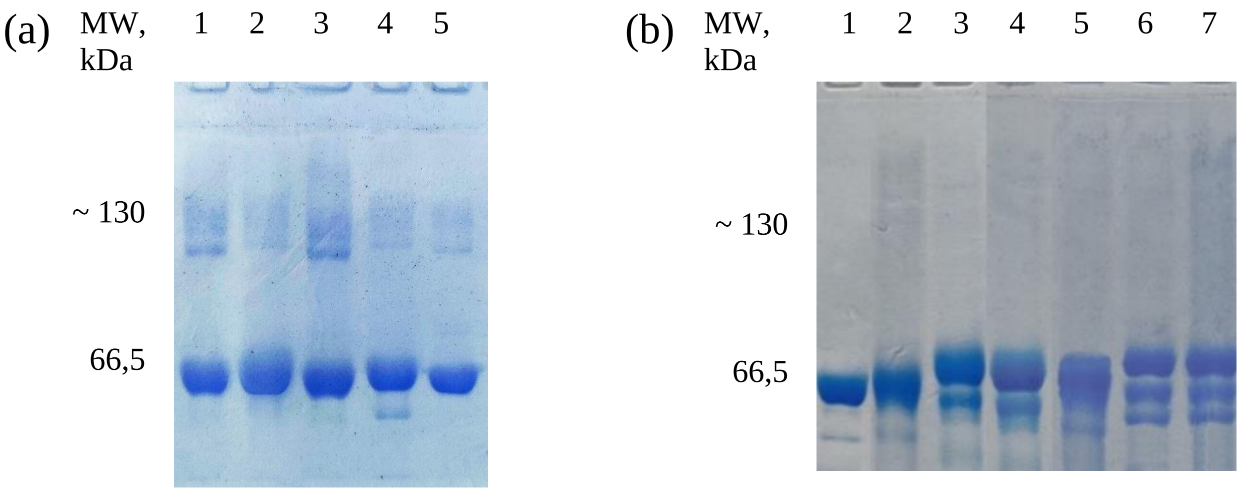

Figure S6. PAGE of HSA-NIT synthesis method 3 under Laemmli condition with the addition of SDS with subsequent Coomassie blue staining after storing samples for 3 months and 1 year. (a) HSA (lane 1); HSA-Me (lane 2); HSA-Me/Me-mal (lane 3); HSA-Et (lane 4); HSA-Et/Et-mal (lane 5). (b) HSA (lane 1); HSA-Me (lane 2); HSA-Me-mal (lane 3); HSA-Me/Me-mal (lane 4); HSA-Et (lane 5); HSA-Et-mal (lane 6); HSA-Et/Et-mal (lane 7).

6. Reduction of HSA-NIT conjugates

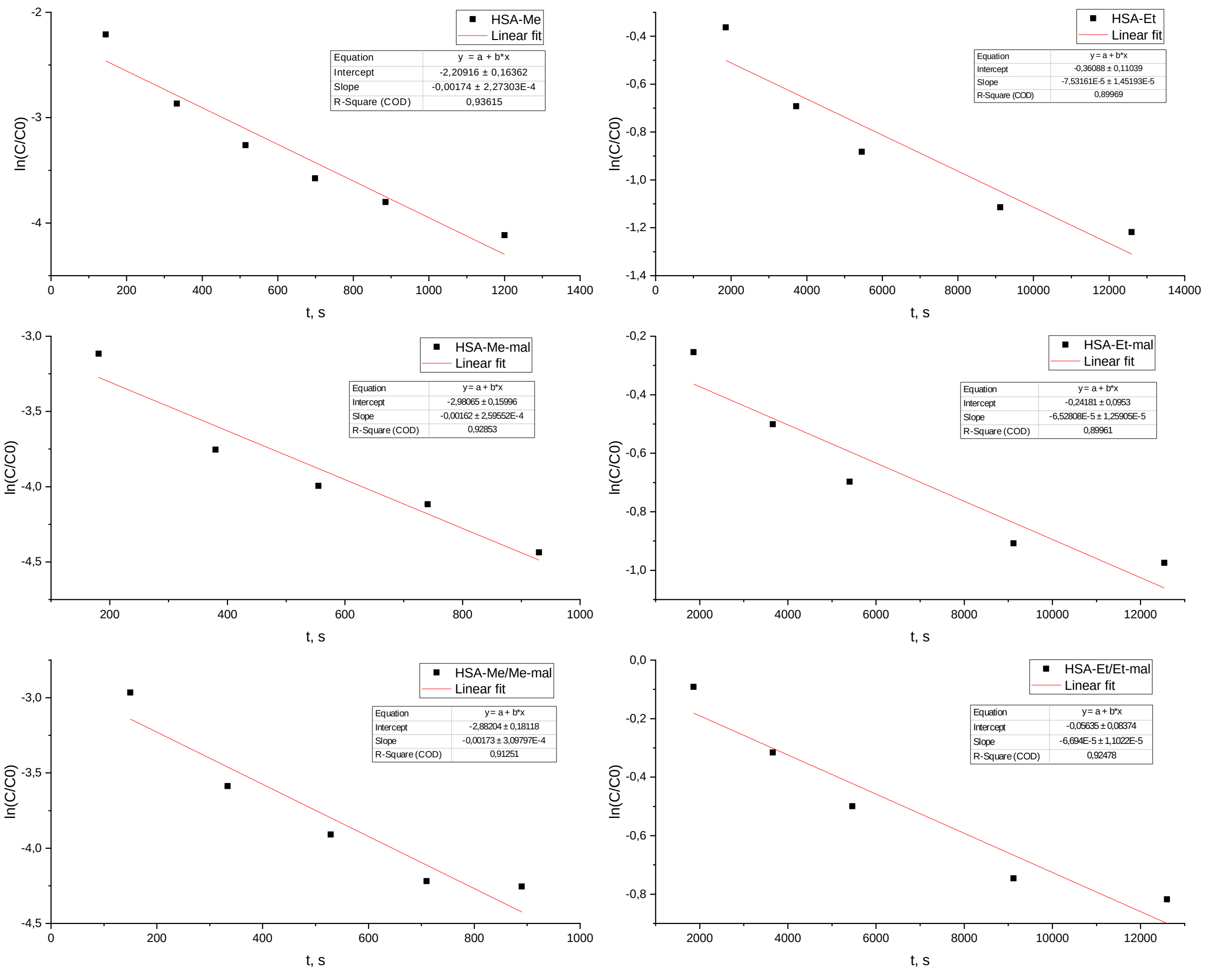

Figure S7. Kinetics of the reduction of HSA-NIT (0.1 mM) by ascorbate (40 mM) /glutathione (20 mM) solution in pH = 7.4 at 25 °C by EPR.

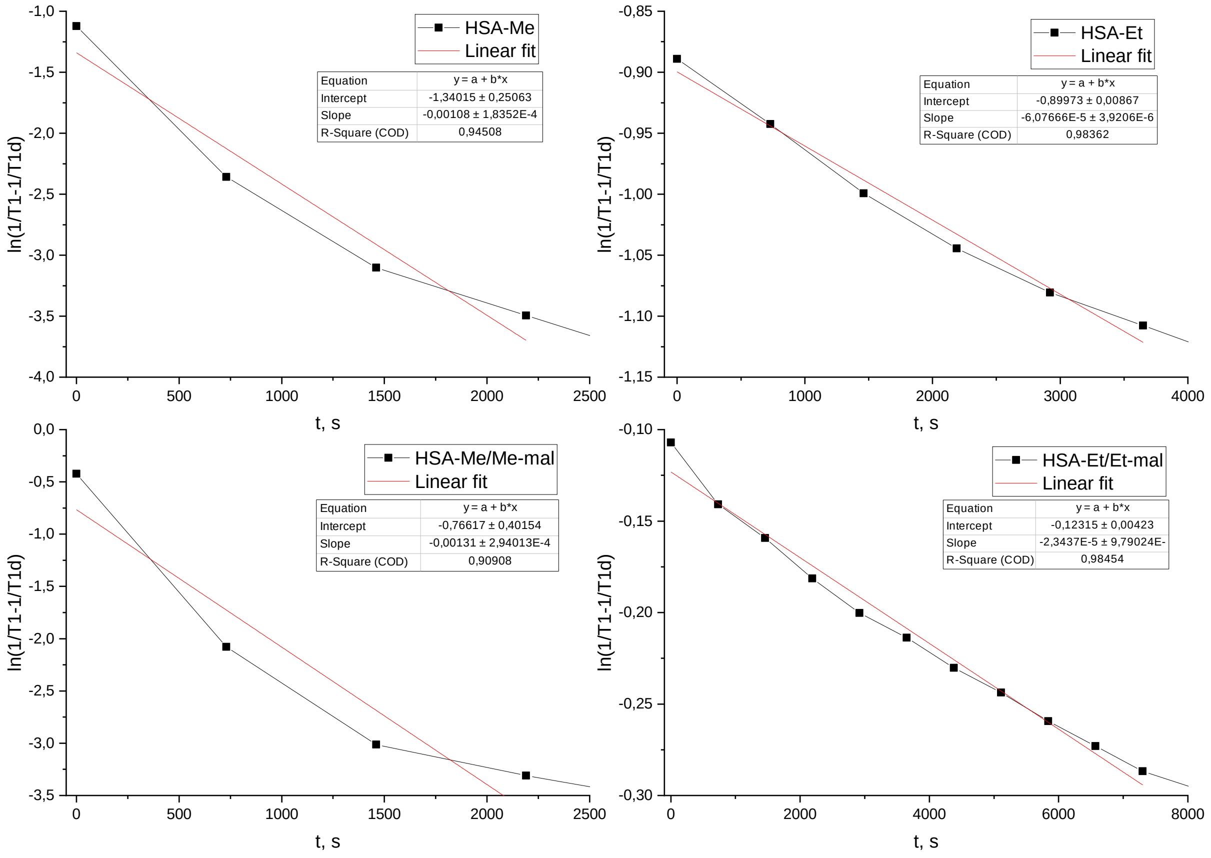

Figure S8. Kinetics of the reduction of HSA-NIT (0.1 mM) by ascorbate (40 mM) /glutathione (20 mM) solution in pH = 7.4 at 25 °C by the method of T_1 relaxation time measuring.

7. Relaxivity r_1 and r_2 and MRI phantoms of HSA-NIT conjugates

Table S4. Relaxivity r_1 and r_2 (mM^{-1}s^{-1}) per HSA molecule for HSA-NIT conjugates at 14.1, 7 T, 3 T, and 1.88 T, 25°C.

Sample	14.1 T			7 T			3 T			1.88 T		
	r_1	r_2	r_2/r_1	r_1	r_2	r_2/r_1	r_1	r_2	r_2/r_1	r_1	r_2	r_2/r_1
HSA-Me	1.59± 0.08	26.3 ± 0.5	16.5	2.76 ± 0.03	18.4 ± 0.2	7.0	3.5 ± 0.1	11.2 ± 0.8	3.2	3.02 ± 0.03	9.1 ± 0.1	3.0
HSA-Me/Me-mal	4.07 ± 0.07	38.2 ± 0.5	9.4	5.16 ± 0.05	34.4 ± 0.5	6.7	8.2 ± 0.1	21.7 ± 0.1	2.6	6.3 ± 0.1	16.9 ± 0.4	2.7
HSA-Me-mal	4.09 ± 0.07	35.9 ± 0.2	8.77	4.98 ± 0.03	33.2 ± 0.1	6.7	8.12 ± 0.09	21.2 ± 0.2	2.6	6.2 ± 0.1	16,6 ± 0.4	2.5
HSA-Et	1.84 ± 0.07	37.8 ± 0.7	20.5	2.96 ± 0.04	29.3 ± 0.3	9.6	4.83 ± 0.09	21.6 ± 0.4	4.5	4.05 ± 0.04	18.0 ± 0.6	4.4
HSA-Et/Et-mal	6.67 ± 0.05	72.6 ± 0.5	10.9	10.04 ± 0.05	96.7 ± 0.3	9.8	15.12 ± 0.05	50.2 ± 0.5	3.3	11.0 ± 0.2	31.6 ± 0.5	2.9
HSA-Et-mal	5.26 ± 0.1	68.7 ± 0.3	13.1	8.76 ± 0.08	84.8 ± 0.2	9.8	11.1 ± 0.1	45.4 ± 0.3	4.1	8,4 ± 0.1	30,1 ± 0.5	3,9

Table S5. Relaxivity r_1 and r_2 for HSA-NIT per NIT at 14.1, 7, 3, and 1.88 T, 25°C.

**Sample**	**14.1 T**		**7 T**		**3 T**		**1.88 T**	
	r_1	r_2	r_1	r_2	r_1	r_2	r_1	r_2
HSA-Me	0.12	1.9	0.20	1.4	0.38	1.2	0.30	0.9
HSA-Me/Me-mal	0.24	2.2	0.30	2.0	0.48	1.3	0.33	0.9
HSA-Me-mal	0.25	2.2	0.30	2.0	0.49	1.3	0.34	0.9
HSA-Et	0.14	2.9	0.23	2.2	0.50	2.1	0.39	1.7
HSA-Et/Et-mal	0.29	3.1	0.43	4.2	0.38	1.3	0.42	1.2
HSA-Et-mal	0.25	3.3	0.42	4.1	0.50	2.0	0.38	1.4

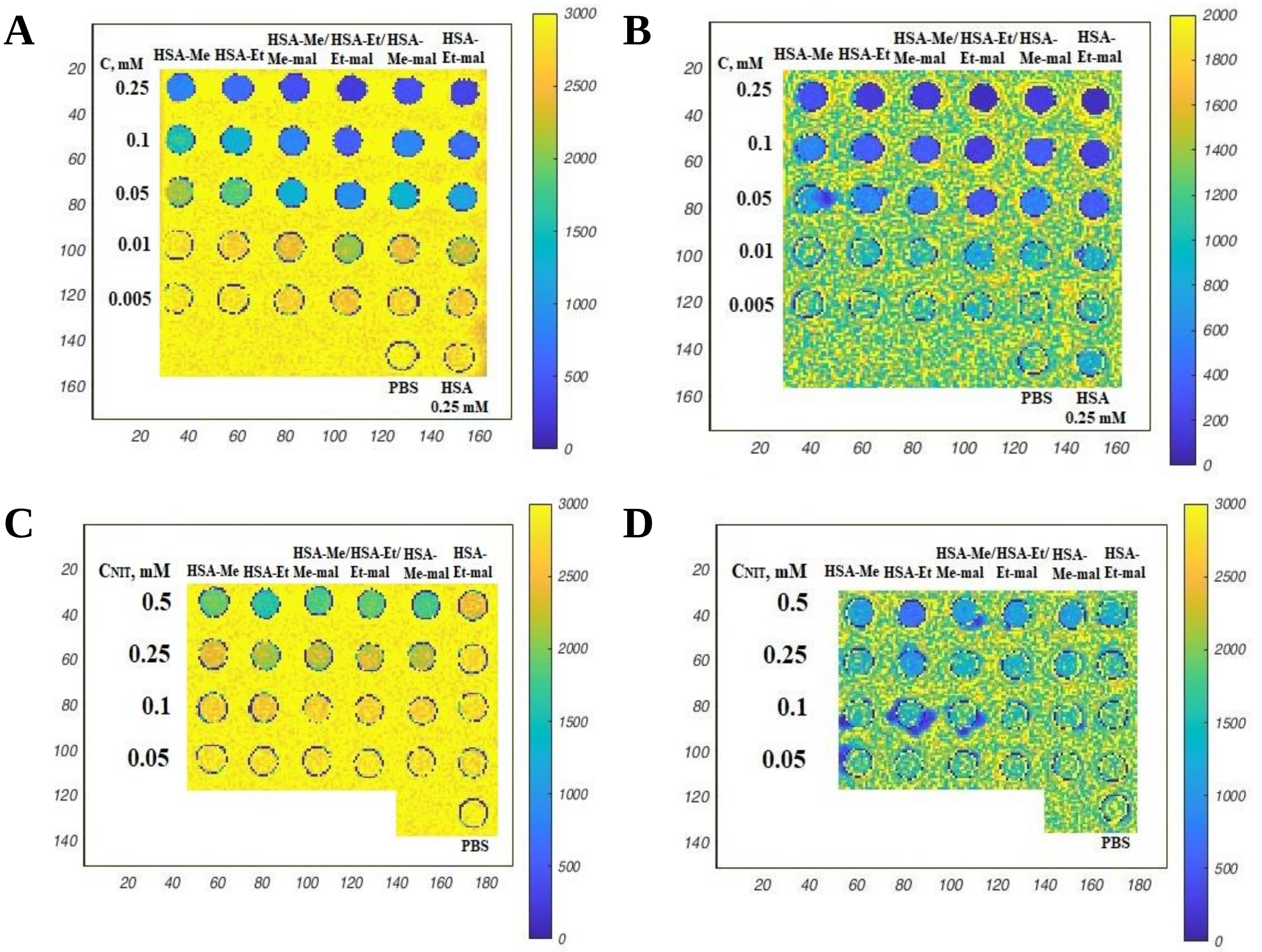

Figure S9. T_1-weighted (A, C) and T_2-weighted (B, D) images of MRI phantoms at indicated HSA-NIT concentrations per HSA (A, B) and per NIT (C, D) imaged at 3 T, 25°C.

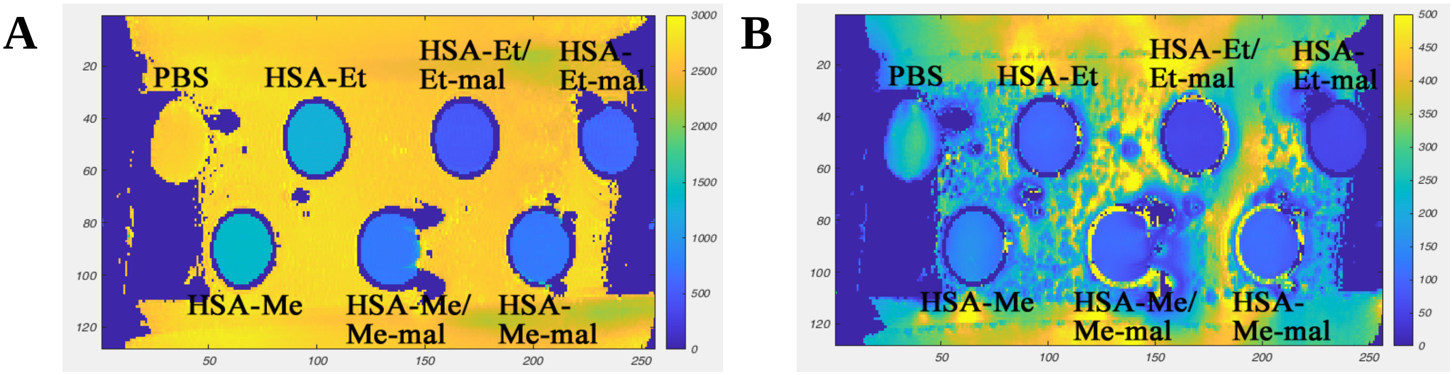

Figure S10. T_1-weighted (A) and T_2-weighted (B) images of MRI phantoms containing 0.25 mM HSA-NIT (concentrations per HSA) imaged at 14.1 T, 25°C.

8. Trypsinolysis of HSA-NIT conjugates

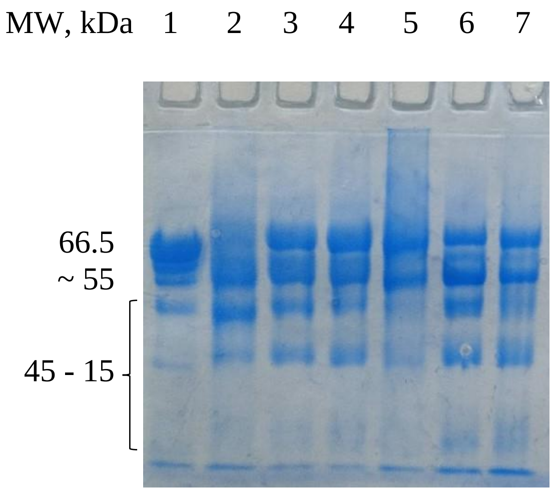

Figure S11. SDS–PAGE of HSA-NIT trypsinolysis under Laemmli condition with subsequent Coomassie blue staining. HSA (lane 1); HSA-Me (lane 2); HSA-Me-mal (lane 3); HSA-Me/Me-mal (lane 4); HSA-Et (lane 5); HSA-Et-mal (lane 6); HSA-Et/Et-mal (lane 7). Trypsinolysis time 1 h.

Table S6. $1/T_1$ and $1/T_2$ values for trypsinolysis of HSA-NIT after 24 h

**Sample**	**Trypsinolysis time 24 h**		**Calculation of possible T values, assuming that all nitroxides are free molecules in the solution***	
	**$1/T_1$, s^{-1}**	**$1/T_2$, s^{-1}**	**$1/T_1$, s^{-1}**	**$1/T_2$, s^{-1}**
HSA-Me	0.69	1.58	0.70	0.90
HSA-Me/Me-mal	1.08	2.51	1.06	1.37
HSA-Et	0.85	2.74	0.79	0.99
HSA-Et/Et-mal	1.51	4.36	1.53	1.89

*r_1(Me) = 0.20 $mM^{-1}s^{-1}$, r_2(Me) = 0.26 $mM^{-1}s^{-1}$, r_1(Et) = 0.24 $mM^{-1}s^{-1}$, and r_2(Et) = 0.29 $mM^{-1}s^{-1}$ were used for calculations. Relaxivities of Me and Et derivatives were measured in the present work by 1.88 T spectrometer.

9. Longitudinal relaxation times at ULF

Table S7. Longitudinal relaxation time $T_1(B)$ at the two different magnetic field strengths B_0 and B_p. Note that the hyperpolarization build-up time $T_{\text{build up}}$ is similar to $T_1(B_p)$.

**Sample**	$\boldsymbol{T_1(B_0)}$	$\boldsymbol{T_{\text{build up}}, T_1(B_p)}$
HSA-Me-mal C	745 ± 8	785 ± 21
HSA-Me/Me-mal C	826 ± 11	816 ± 29
HSA-Me/Me-mal T	736 ± 18	811 ± 12
HSA-Et-mal C	643 ± 17	666 ± 28
HSA-Et/Et-mal C	897 ± 25	938 ± 32
HSA-Et/Et-mal T	868 ± 19	927 ± 23

* The sample name has been extended to include the type of protease, either C or T, for which the reaction kinetics were monitored.

10. Reproducibility of real-time trypsin-mediated cleavage tracking

Due to the limited amount of samples, it was not possible to perform multiple measurements of the trypsin-mediated cleavage process for all samples. Nevertheless, we successfully monitored the kinetics of the cleavage process on three occasions for the HSA-Et/Et-mal sample (see Figure S12). It is noteworthy that these measurements were performed at room temperature, which may account for the slight discrepancy in reaction rates compared to those reported in the main manuscript (performed at 37°C). The fitting parameters for Equation 1 are shown in Figure S12 and indicate an accuracy of about 10% over the three measurements.

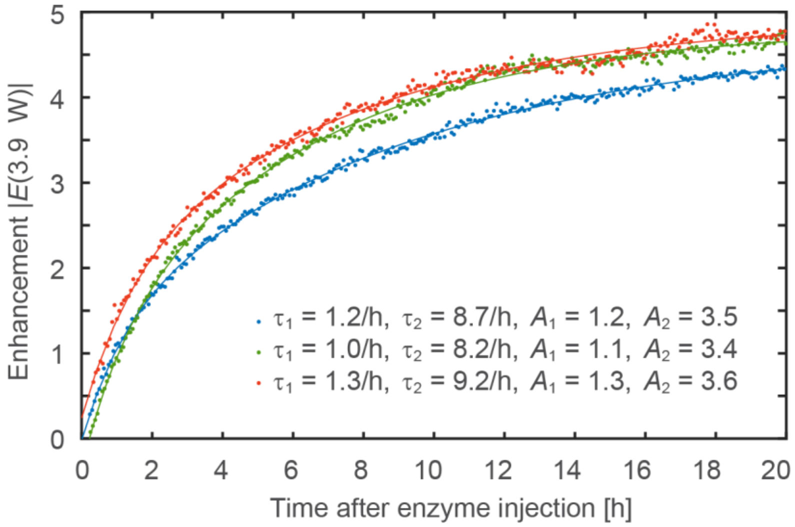

Figure S12. Reproducibility of $E(t)$ measurements for HSA-Et/Et-mal after injection of trypsin at room temperature.
